# Generalized nonlinear Schrödinger equations describing the Second Harmonic Generation of femtosecond pulse, containing a few cycles, and their integrals of motion

**DOI:** 10.1371/journal.pone.0226119

**Published:** 2019-12-12

**Authors:** Vyacheslav A. Trofimov, Svetlana Stepanenko, Alexander Razgulin

**Affiliations:** Faculty of Computational Mathematics and Cybernetics, Lomonosov Moscow State University, Moscow, Russia; China University of Mining and Technology, CHINA

## Abstract

An interaction of laser pulse, containing a few cycles, with substance is a modern problem, attracting attention of many researches. The frequency conversion is a key problem for a generation of such pulses in various ranges of frequencies. Adequate description of such pulse interaction with a medium is based on a slowly evolving wave approximation (SEWA), which has been proposed earlier for a description of propagation of the laser pulse, containing a few cycles, in a medium with cubic nonlinear response. Despite widely applicability of the frequency conversion for various nonlinear optics problems solutions, SEWA has not been applied and developed for a theoretical investigation of the frequency doubling process until present time. In this study the set of generalized nonlinear Schrödinger equations describing a second harmonic generation of the super-short femtosecond pulse is derived. The equations set contains terms, describing the pulses self-steepening, and the second order dispersion (SOD) of the pulse, a diffraction of the beam as well as mixed derivatives. We propose the transform of the equations set to a type, which does not contain both the mixed derivatives and time derivatives of the nonlinear terms. This transform allows us to derive the integrals of motion of the problem: energy, spectral invariants and Hamiltonian. We show the existence of two specific frequencies (singularities in the Fourier space) inherent to the problem. They may cause an appearance of non-physical absolute instability of the problem solution if the spectral invariants are not taken into account. Moreover, we claim that the energy preservation at the laser pulses propagation may not occur if these invariants do not preserve. Developed conservation laws, in particular, have to be used for developing of the conservative finite-difference schemes, preserving the conservation laws difference analogues, and for developing of adequate theory of the modulation instability of the laser pulses, containing a few cycles.

## Introduction

A well-known process of second harmonic generation (SHG) by the laser beam was the first nonlinear optical effect, which was observed by P. Franken et al. [1] in 1961. One year later, J. Armstrong, N. Bloembergen et al. [2] published the fundamental paper, dealing with the optical frequency conversion, in which the coupled nonlinear equations were developed and many explicit solutions of these equations were obtained in the framework of the plane wave approximation. During passed decades the SHG was widely observed: in plasma optics [3–5] and nonlinear optics, including high-harmonic generation [6–12], and in a medium containing nanoparticles [13, 14], and in semiconductor [15]. In [16] the SHG at the boundary between dielectric media is analyzed. Obviously, this list of papers can be easily increased.

Many researchers pay an attention to an investigation of various factors, which restrict the frequency doubling efficiency. For example, in [17] the effect of a group-velocity dispersion was considered. In [18] the authors have considered an influence of interplaying between two types polarizations of the pulse at the fundamental frequency on the frequency doubling efficiency. With increasing of the laser pulse intensity it is necessary to take into account a cubic nonlinear response of a medium under the SHG. An influence of the self-modulation as well as cross-modulation of the laser pulse on the frequency doubling efficiency was investigated experimentally and theoretically (see, for example [2, 19–28]). It should be stressed that the most general analytical theory of this process has been developed recently in [28], taking into account the time-dependent pulse shape and without using the basic wave energy non-depletion approximation on the base of using the integrals of motion (conservation laws) of the problem. We notice that the authors of [22] have obtained the most (but not all) solutions of this problem in the framework of long pulse duration approximation. In [29] an effective way for increasing of SHG efficiency in bulk medium was demonstrated by using of the incident beam with a ring (tubular) profile. This leads to re-profiling of the beams due to their diffraction and, therefore, to decreasing the beam phase distortions.

New opportunities appeared at SHG under the big phase-mismatching, so-called cascading SHG [30–33]. This process allows us to achieve an effective cubic nonlinear response in a medium with a quadratic nonlinear response. This effect was firstly predicted by Yu. N. Karamzin and A. P. Sukhorukov [34] in 1974. Later, it was used for a compression of the soliton with femtosecond duration [30, 34–50], and for the beam self-focusing [51–53], and for using the cascading SHG for suppression of the optical pulse intensity fluctuations, occurring in a sequence of pulses with randomly variations of their maximal intensities [54–57]. Such sequence of the laser pulses is produced by a laser, operating at the free generation mode. The total duration of the pulses sequences is equal to milliseconds. Therefore, the cascading SHG allows realizing the laser generation mode that is similar to the Kerr-locked mode applying for the femtosecond laser system.

There are a lot of other schemes of SHG, using the modern substances (see, for example, [58–84]) and at a present time the problem is still actual and important for various applications. Among these applications, we stress using of SHG for a measurement of pulse parameters and of parameters of a medium as well as for visualization of various fast processes, occurring in the substances. The most famous approaches of parameters measuring are SPIDER (spectral interferometry for direct electric-field reconstruction) [85] and FROG (frequency-resolved optical gating) [86, 87]. Also, in [88], a technique, based on combination of FROG spectra measurements was proposed to completely characterize the amplitude and phase of an ultrashort pulse in space and time. Mix FROG method was implemented in [89]. Thus, the frequency doubling of the optical pulse, containing a few cycles, is of practical interest until present time. However, in this case it is necessary to take into account the self-steepening of the laser pulses on both fundamental and doubled frequencies. According to our knowledge, the first analysis of this problem was made numerically in [90] at the group velocity synchronism and phase matching, without taking into account the beam diffraction and the second order dispersion influence on the pulses interaction. For writing the corresponding equations, the authors have applied an approach, which was developed first by N. Tzoar and M. Jain [91] under the investigation of the single pulse propagation in a medium with a cubic nonlinear response. Experimental observation of the self-steepening influence on the pulse spectrum deformation was demonstrated in [92]. Then, this approach was successfully used for describing the processes of a laser pulse propagation in the optical fiber [93]. The corresponding nonlinear Schrödinger equation (NLSE) contains the first time derivative of a nonlinear medium response. This equation was derived using the slowly varying envelope approximation (SVEA) and named as generalized NLSE.

Taking into account a laser beam diffraction, T. Brabec and F. Krausz proposed in 1997 a new approach, so-called slowly evolving wave approximation (SEWA). Derived equation was also named as the generalized nonlinear Schrödinger equation (GNLSE) [94]. Let us note that the SEWA requires not only an envelope of a wave packet, but also the phase changing must slowly vary as the pulse covers a distance, which is equal to the wavelength. The main feature of GNLSE consists in its containing of mixed derivatives on time and spatial coordinates, and of the second-order time derivative of a medium nonlinear response (which describes the pulse self-steepening).

It should be emphasized that a differential operator of the GNLSE, written in [91, 93], coincides with a differential operator of the GNLSE, derived in [94] if the optical beam diffraction does not take into account. They differ only by the factor two at the coefficient characterizing the self-steepening term of the equation. Obviously, these equations written in the dimensionless units look the same.

Finding of conservation laws attracts an attention of various authors (see, for example, recently published papers [95–99]). In papers [100, 101] we derived the conservation laws for the frequency doubling process if the self-steepening of the pulses occurs. However, until present time, the set of GNLSEs describing a SHG of super-short femtosecond pulse with taking into account a diffraction of the laser beam (it means that this process is described in the framework of the SEWA) as well as their conservation laws were not derived. We do this in the present paper.

As is well-known, the invariants are very useful for computer simulation of the laser pulse propagation because the equations describing various problems of nonlinear optics are nonlinear ones and as a rule they do not allow us to find their analytical solution. As a consequence, the finite-difference time-domain (FDTD) method [102, 103] for computer simulation of a propagation of the optical pulse, containing a few cycles, is usually employed. For example, in [104] the authors develop the time-transformation method based on FDTD. An alternative method, which permits applying fast numerical algorithms, is a generalized source method (GSM) [105]. Linear GSM was adapted to describe the SHG in diffraction gratings, containing non-centrosymmetric materials [106]. Obviously, there are other papers, in which a computer simulation is applied for an investigation of the SHG problem. As a rule, a split-step method was used at computer simulation.

Another approach for computer simulation is based on developing of the conservative finite-difference schemes [107]. They allow preserving the difference analogues of the corresponding conservation laws. Obviously, it is necessary firstly to write these conservation laws. With this aim, we propose a new transform for the derived GNLSEs, which reduces the equations to the form containing neither mixed derivatives on time and spatial coordinates nor time derivatives of the nonlinear response of a medium. Using a new set of GNLSEs, we obtain the energy invariant, spectral invariants and Hamiltonian. We add some specific requirements to the problem statement (excluding of the singularities in frequency space) to avoid a development of non-physical instability of the SHG process at taking into account the pulses self-steepening. These requirements, together with the spectral invariants, are also important for a validity of the energy conservation law.

## Generalized nonlinear Schrödinger equations derivation

### Derivation of the equations for SHG process in the framework of SEWA

Derivation of the equations describing the optical frequency doubling in the framework of SEWA for an electric field strength *E*(*z*, *x*, *t*) starts from the well-known wave equation:

$$\begin{array}{c} \left(\partial_{z}^{2}+\nabla_{⊥}^{2}\right)E\left(z,x,t\right)-\frac{1}{c^{2}}\partial_{t}^{2}D\left(z,x,t\right)=0, \end{array}$$ (1)

written above in 2*D* case, for example. The laser pulse propagates along *z* coordinate, *t* is a time, *c* is the light velocity in a vacuum, *x* is a transverse coordinate to the optical pulse propagation direction, an operator $\nabla_{⊥}^{2}=\partial_{x}^{2}$ is the transversal Laplace operator. We choose only *x*-coordinate as a transverse one for simplicity. This does not restrict our consideration. The function *D*(*z*, *x*, *t*) describes an electric field induction and is written in the form:

$$\begin{array}{c} D(z,x,t)=E(z,x,t)+4\pi P(z,x,t), \end{array}$$ (2)

the function *P*(*z*, *x*, *t*) is a medium polarization, containing its linear and nonlinear responses:

$$\begin{array}{c} P\left(z,x,t\right)=P_{lin}\left(z,x,t\right)+P_{nl}\left(z,x,t\right). \end{array}$$ (3)

For linear non-instantaneous medium response *P*_*lin*_(*z*, *x*, *t*) the following representation

$$\begin{array}{c} P_{lin}\left(z,x,t\right)=\int_{0}^{+\infty }\chi^{\left(1\right)}\left(t^{\prime }\right)E\left(z,x,t-t^{\prime }\right)dt^{\prime } \end{array}$$ (4)

is widely used, which yields in the relation for a dielectric permittivity:

$$\begin{array}{c} \varepsilon \left(t\right)=\frac{1}{2\pi }\int_{-\infty }^{+\infty }\tilde{\varepsilon }\left(\omega \right)\exp\left(-i\omega t\right)d\omega ,\;\tilde{\varepsilon }\left(\omega \right)=1+4\pi {\tilde{\chi }}^{\left(1\right)}\left(\omega \right), \end{array}$$ (5)

here ${\tilde{\chi }}^{\left(1\right)}\left(\omega \right)$ is a linear electric susceptibility of a medium at the frequency *ω*.

Function *P*_*nl*_(*z*, *x*, *t*) describes a nonlinear response of a medium. If we consider a medium with the quadratic nonlinearity, then this function is defined as *P*_*nl*_ = *χ*^(2)^*E*^2^, and *χ*^(2)^ is a quadratic electric susceptibility.

The next step for the wave equation reduction consists in representation of an electric field strength *E*(*z*, *x*, *t*) in a following manner:

$$\begin{array}{c} E\left(z,x,t\right)=E_{1}\left(z,x,t\right)+E_{2}\left(z,x,t\right)=\frac{1}{2}(A_{1}\left(z,x,t\right)\exp\left[-i\left(\omega_{o}t-k_{1}z\right)\right]+\\ +A_{2}\left(z,x,t\right)\exp\left[-i\left(2\omega_{o}t-k_{2}z\right)\right]+c.c.), \end{array}$$ (6)

where *E*_1_(*z*, *x*, *t*), *E*_2_(*z*, *x*, *t*) are the electric field strengths of waves, propagating with carrier frequency *ω*_*o*_, and with doubled frequency 2*ω*_*o*_ and with wave-numbers *k*_1_, *k*_2_, correspondingly. In (6) we introduce the slowly varying envelopes for both wave packets and denote them as *A*_1_(*z*, *x*, *t*) and *A*_2_(*z*, *x*, *t*). Symbols *c*.*c*. denote a complex conjugation. The linear polarization of a medium at these frequencies can be written as

$$\begin{array}{c} P_{lin}\left(z,x,t\right)=P_{{lin}_{1}}\left(z,x,t\right)+P_{{lin}_{2}}\left(z,x,t\right)=\frac{1}{2}(L_{1}\left(z,x,t\right)\exp\left[-i\left(\omega_{o}t-k_{1}z\right)\right]+\\ +L_{2}\left(z,x,t\right)\exp\left[-i\left(2\omega_{o}t-k_{2}z\right)\right]+c.c.). \end{array}$$ (7)

The corresponding nonlinear polarization of a medium at chosen frequencies is written as follows

$$\begin{array}{c} P_{nl}\left(z,x,t\right)=\frac{\chi^{\left(2\right)}}{4}(A_{1}^{2}\exp\left[-i\left(2\omega_{o}t-\left(2k_{1}-k_{2}\right)z-k_{2}z\right)\right]+\\ +2A_{1}^{*}A_{2}\exp\left[-i\left(\omega_{o}t+\left(2k_{1}-k_{2}\right)z-k_{1}z\right)\right]+c.c.). \end{array}$$ (8)

Let us remind that the following relations for wave-numbers are valid:

$$\begin{array}{cc} k_{1}=n_{\omega }\left(\omega_{o}/c\right) & =\sqrt{\tilde{\varepsilon }\left(\omega_{o}\right)}\left(\omega_{o}/c\right),\\ k_{2}=n_{2\omega }\left(2\omega_{o}/c\right) & =\sqrt{\tilde{\varepsilon }\left(2\omega_{o}\right)}\left(2\omega_{o}/c\right), \end{array}$$

where *n*_*ω*_, *n*_2*ω*_ are the medium refractive indexes at the frequencies *ω*_*o*_ and 2*ω*_*o*_, correspondingly.

Taking into account the expressions (6) and (8) and (1) transforms to the following form:

$$\begin{array}{c} \frac{1}{2}\exp\left(ik_{1}z\right)[(\frac{\partial^{2}A_{1}}{\partial z^{2}}+2ik_{1}\frac{\partial A_{1}}{\partial z}-k_{1}^{2}A_{1}+\frac{\partial^{2}A_{1}}{\partial x^{2}})\exp\left(-i\omega_{o}t\right)-\\ -\frac{1}{c^{2}}\partial_{t}^{2}\left(D_{1}\left(z,x,t\right)+4\pi \chi^{\left(2\right)}A_{1}^{*}A_{2}\exp\left[-i\left(\omega_{o}t+\left(2k_{1}-k_{2}\right)z\right)\right]\right)]+\\ +\frac{1}{2}\exp\left(ik_{2}z\right)[(\frac{\partial^{2}A_{2}}{\partial z^{2}}+2ik_{2}\frac{\partial A_{2}}{\partial z}-k_{2}^{2}A_{2}+\frac{\partial^{2}A_{2}}{\partial x^{2}})\exp\left(-2i\omega_{o}t\right)-\\ -\frac{1}{c^{2}}\partial_{t}^{2}\left(D_{2}\left(z,x,t\right)+2\pi \chi^{\left(2\right)}A_{1}^{2}\exp\left[-i\left(2\omega_{o}t-\left(2k_{1}-k_{2}\right)z\right)\right]\right)]+c.c.=0, \end{array}$$ (9)

where *D*_*j*_(*z*, *x*, *t*), *j* = 1, 2 is a linear part of the electric field induction at the frequencies *ω*_*o*_ and 2*ω*_*o*_, correspondingly:

$$\begin{array}{c} D_{j}\left(z,x,t\right)=\left(A_{j}\left(z,x,t\right)+4\pi L_{j}\left(z,x,t\right)\right)\exp\left(-ij\omega_{o}t\right). \end{array}$$ (10)

Let us multiply (9) by exp(− *ik*_1_*z*) or exp(− *ik*_2_*z*). Then, we integrate each of these expressions over the corresponding wave period, taking into account the orthogonal property of the sine and cosine functions. Then, (9) can be reduced to the following equations:

$$\begin{array}{c} (\frac{\partial^{2}A_{1}}{\partial z^{2}}+2ik_{1}\frac{\partial A_{1}}{\partial z}-k_{1}^{2}A_{1}+\frac{\partial^{2}A_{1}}{\partial x^{2}}+\\ +\frac{4\pi \chi^{\left(2\right)}\omega_{o}^{2}}{c^{2}}(1+\frac{i}{\omega_{o}}\frac{\partial }{\partial t}{)}^{2}A_{1}^{*}A_{2}\exp\left[-i\left(2k_{1}-k_{2}\right)z\right])\exp\left(-i\omega_{o}t\right)-\\ -\frac{1}{c^{2}}\partial_{t}^{2}D_{1}\left(z,x,t\right)=0,\\ (\frac{\partial^{2}A_{2}}{\partial z^{2}}+2ik_{2}\frac{\partial A_{2}}{\partial z}-k_{2}^{2}A_{2}+\frac{\partial^{2}A_{2}}{\partial x^{2}}+\\ +\frac{2\pi \chi^{\left(2\right)}{\left(2\omega_{o}\right)}^{2}}{c^{2}}(1+\frac{i}{2\omega_{o}}\frac{\partial }{\partial t}{)}^{2}A_{1}^{2}\exp\left[i\left(2k_{1}-k_{2}\right)z\right])\exp\left(-2i\omega_{o}t\right)-\\ -\frac{1}{c^{2}}\partial_{t}^{2}D_{2}\left(z,x,t\right)=0. \end{array}$$ (11)

Let us note the Fourier transform property:

$$\begin{array}{c} \partial_{t}^{2}D_{j}\left(z,x,t\right)=\frac{1}{2\pi }\partial_{t}^{2}\int_{-\infty }^{+\infty }\tilde{D_{j}}\left(z,x,\omega \right)\exp\left(-i\omega t\right)d\omega =\\ =-\frac{1}{2\pi }\int_{-\infty }^{+\infty }\omega^{2}\tilde{D_{j}}\left(z,x,\omega \right)\exp\left(-i\omega t\right)d\omega ,\;j=1,2. \end{array}$$ (12)

Therefore, (11) can be written as:

$$\begin{array}{c} (\frac{\partial^{2}A_{1}}{\partial z^{2}}+2ik_{1}\frac{\partial A_{1}}{\partial z}-k_{1}^{2}A_{1}+\frac{\partial^{2}A_{1}}{\partial x^{2}}+\\ +\frac{4\pi \chi^{\left(2\right)}\omega_{o}^{2}}{c^{2}}(1+\frac{i}{\omega_{o}}\frac{\partial }{\partial t}{)}^{2}A_{1}^{*}A_{2}\exp\left[-i\left(2k_{1}-k_{2}\right)z\right])\exp\left(-i\omega_{o}t\right)+\\ +\frac{1}{2\pi }\exp\left(-i\omega_{o}t\right)\int_{-\infty }^{+\infty }\frac{\omega^{2}}{c^{2}}\tilde{\varepsilon }\left(\omega \right)\tilde{A_{1}}\left(z,x,\omega -\omega_{o}\right)\exp\left[-i\left(\omega -\omega_{o}\right)t\right]d\omega =0,\\ (\frac{\partial^{2}A_{2}}{\partial z^{2}}+2ik_{2}\frac{\partial A_{2}}{\partial z}-k_{2}^{2}A_{2}+\frac{\partial^{2}A_{2}}{\partial x^{2}}-\\ +\frac{2\pi \chi^{\left(2\right)}{\left(2\omega_{o}\right)}^{2}}{c^{2}}(1+\frac{i}{2\omega_{o}}\frac{\partial }{\partial t}{)}^{2}A_{1}^{2}\exp\left[i\left(2k_{1}-k_{2}\right)z\right])\exp\left(-2i\omega_{o}t\right)+\\ +\frac{1}{2\pi }\exp\left(-2i\omega_{o}t\right)\int_{-\infty }^{+\infty }\frac{\omega^{2}}{c^{2}}\tilde{\varepsilon }\left(\omega \right)\tilde{A_{2}}\left(z,x,\omega -2\omega_{o}\right)\exp\left[-i\left(\omega -2\omega_{o}\right)t\right]d\omega =\\ =0. \end{array}$$ (13)

Taking into account the dispersion relation:

$$\begin{array}{c} k^{2}\left(\omega \right)=\frac{\omega^{2}}{c^{2}}\tilde{\varepsilon }\left(\omega \right), \end{array}$$ (14)

one can write (13) as follows

$$\begin{array}{c} (\frac{\partial^{2}A_{1}}{\partial z^{2}}+2ik_{1}\frac{\partial A_{1}}{\partial z}-k_{1}^{2}A_{1}+\frac{\partial^{2}A_{1}}{\partial x^{2}}+\\ +\frac{4\pi \chi^{\left(2\right)}\omega_{o}^{2}}{c^{2}}(1+\frac{i}{\omega_{o}}\frac{\partial }{\partial t}{)}^{2}A_{1}^{*}A_{2}\exp\left[-i\left(2k_{1}-k_{2}\right)z\right])\exp\left(-i\omega_{o}t\right)+\\ +\frac{1}{2\pi }\exp\left(-i\omega_{o}t\right)\int_{-\infty }^{+\infty }k^{2}\left(\omega \right)\tilde{A_{1}}\left(z,x,\omega -\omega_{o}\right)\exp\left[-i\left(\omega -\omega_{o}\right)t\right]d\omega =0, \end{array}$$ (15)

$$\begin{array}{c} (\frac{\partial^{2}A_{2}}{\partial z^{2}}+2ik_{2}\frac{\partial A_{2}}{\partial z}-k_{2}^{2}A_{2}+\frac{\partial^{2}A_{2}}{\partial x^{2}}+\\ +\frac{2\pi \chi^{\left(2\right)}{\left(2\omega_{o}\right)}^{2}}{c^{2}}(1+\frac{i}{2\omega_{o}}\frac{\partial }{\partial t}{)}^{2}A_{1}^{2}\exp\left[i\left(2k_{1}-k_{2}\right)z\right])\exp\left(-2i\omega_{o}t\right)+\\ +\frac{1}{2\pi }\exp\left(-2i\omega_{o}t\right)\int_{-\infty }^{+\infty }k^{2}\left(\omega \right)\tilde{A_{2}}\left(z,x,\omega -2\omega_{o}\right)\exp\left[-i\left(\omega -2\omega_{o}\right)t\right]d\omega =\\ =0. \end{array}$$ (16)

Normally, we expand *k*(*ω*) in a series near the frequency *ω*_*o*_ in (15):

$$\begin{array}{c} k^{2}\left(\omega \right)=(k_{1}+\sum_{n=1}^{\infty }\frac{\partial^{\left(n\right)}k\left(\omega \right)}{\partial \omega^{n}}{|}_{\omega =\omega_{o}}\frac{{\left(\omega -\omega_{o}\right)}^{n}}{n!}{)}^{2}=\\ =(k_{1}+\beta_{1}\left(\omega -\omega_{o}\right)+\frac{\beta_{2}}{2}{\left(\omega -\omega_{o}\right)}^{2}+\sum_{n=3}^{\infty }\frac{\beta_{n}{\left(\omega -\omega_{o}\right)}^{n}}{n!}{)}^{2} \end{array}$$ (17)

and near the frequency 2*ω*_*o*_ in (16):

$$\begin{array}{c} k^{2}\left(\omega \right)=(k_{2}+\sum_{m=1}^{\infty }\frac{\partial^{\left(m\right)}k\left(\omega \right)}{\partial \omega^{m}}{|}_{\omega =2\omega_{o}}\frac{{\left(\omega -2\omega_{o}\right)}^{m}}{m!}{)}^{2}=\\ =(k_{2}+s_{1}\left(\omega -2\omega_{o}\right)+\frac{s_{2}}{2}{\left(\omega -2\omega_{o}\right)}^{2}+\sum_{m=1}^{\infty }\frac{s_{m}{\left(\omega -2\omega_{o}\right)}^{m}}{m!}{)}^{2}, \end{array}$$ (18)

here *β*_*n*_ and *s*_*m*_ are the *n*–th or *m*–th order derivatives of the wave number at the frequencies *ω*_*o*_ and 2*ω*_*o*_, correspondingly.

Let us neglect the terms *β*_*n*_, *s*_*m*_, *n*, *m* = 3, 4, … referring to the third order dispersion and other higher terms in (17) and (18). Also, we neglect the term (*ω* − *ω*_*o*_)^4^. Thus, (17) and (18) take the forms:

$$\begin{array}{c} k^{2}\left(\omega \right)=k_{1}^{2}+2k_{1}\beta_{1}\left(\omega -\omega_{o}\right)+k_{1}\beta_{2}{\left(\omega -\omega_{o}\right)}^{2}+\beta_{1}^{2}{\left(\omega -\omega_{o}\right)}^{2}+\beta_{1}\beta_{2}{\left(\omega -\omega_{o}\right)}^{3}, \end{array}$$ (19)

$$\begin{array}{c} k^{2}\left(\omega \right)=k_{2}^{2}+2k_{2}s_{1}\left(\omega -2\omega_{o}\right)+k_{2}s_{2}{\left(\omega -2\omega_{o}\right)}^{2}+\\ +s_{1}^{2}{\left(\omega -2\omega_{o}\right)}^{2}+s_{1}s_{2}{\left(\omega -2\omega_{o}\right)}^{3}. \end{array}$$ (20)

After Inverse Fourier transform, we obtain the following equations set for describing the frequency doubling process:

$$\begin{array}{c} \left({\left(ik_{1}+\frac{\partial }{\partial z}\right)}^{2}+\frac{\partial^{2}}{\partial x^{2}}\right)A_{1}+\\ +(k_{1}^{2}+2ik_{1}\beta_{1}\frac{\partial }{\partial t}-k_{1}\beta_{2}\frac{\partial^{2}}{\partial t^{2}}-\beta_{1}^{2}\frac{\partial^{2}}{\partial t^{2}}-i\beta_{1}\beta_{2}\frac{\partial^{3}}{\partial t^{3}})A_{1}+\\ +\frac{4\pi \omega_{o}^{2}\chi^{\left(2\right)}}{c^{2}}(1+\frac{i}{\omega_{o}}\frac{\partial }{\partial t}{)}^{2}A_{1}^{*}A_{2}\exp\left(i\Delta kz\right)=0, \end{array}$$ (21)

$$\begin{array}{c} \left({\left(ik_{2}+\frac{\partial }{\partial z}\right)}^{2}+\frac{\partial^{2}}{\partial x^{2}}\right)A_{2}+\\ +(k_{2}^{2}+2ik_{2}s_{1}\frac{\partial }{\partial t}-k_{2}s_{2}\frac{\partial^{2}}{\partial t^{2}}-s_{1}^{2}\frac{\partial^{2}}{\partial t^{2}}-is_{1}s_{2}\frac{\partial^{3}}{\partial t^{3}})A_{2}+\\ +\frac{2\pi {\left(2\omega_{o}\right)}^{2}\chi^{\left(2\right)}}{c^{2}}(1+\frac{i}{2\omega_{o}}\frac{\partial }{\partial t}{)}^{2}A_{1}^{2}\exp\left(-i\Delta kz\right)=0. \end{array}$$ (22)

Above Δ*k* = *k*_2_ − 2*k*_1_ is the phase mismatch. (21) and (22) are called as the set of generalized nonlinear Schrödinger equations (GNLSEs).

### Coordinate system transform

#### First type of coordinate system transform

In a coordinate system moving with the first wave packet

$$\begin{array}{c} \xi =z,\;\tau =t-\beta_{1}z, \end{array}$$ (23)

(21) and (22) could be re-written as follows:

$$\begin{array}{c} ((ik_{1}+\frac{\partial }{\partial \xi }-\beta_{1}\frac{\partial }{\partial \tau }{)}^{2}+\frac{\partial^{2}}{\partial x^{2}})A_{1}+\\ +(k_{1}^{2}+2ik_{1}\beta_{1}\frac{\partial }{\partial \tau }-k_{1}\beta_{2}\frac{\partial^{2}}{\partial \tau^{2}}-\beta_{1}^{2}\frac{\partial^{2}}{\partial \tau^{2}}-i\beta_{1}\beta_{2}\frac{\partial^{3}}{\partial \tau^{3}})A_{1}+\\ +\frac{4\pi {\left(\omega_{o}\right)}^{2}\chi^{\left(2\right)}}{c^{2}}(1+\frac{i}{\omega_{o}}\frac{\partial }{\partial \tau }{)}^{2}A_{1}^{*}A_{2}\exp\left(i\Delta k\xi \right)=0, \end{array}$$ (24)

$$\begin{array}{c} ((ik_{2}+\frac{\partial }{\partial \xi }-\beta_{1}\frac{\partial }{\partial \tau }{)}^{2}+\frac{\partial^{2}}{\partial x^{2}})A_{2}+\\ +(k_{2}^{2}+2ik_{2}s_{1}\frac{\partial }{\partial \tau }-k_{2}s_{2}\frac{\partial^{2}}{\partial \tau^{2}}-s_{1}^{2}\frac{\partial^{2}}{\partial \tau^{2}}-is_{1}s_{2}\frac{\partial^{3}}{\partial \tau^{3}})A_{2}+\\ +\frac{2\pi {\left(2\omega_{o}\right)}^{2}\chi^{\left(2\right)}}{c^{2}}(1+\frac{i}{2\omega_{o}}\frac{\partial }{\partial \tau }{)}^{2}A_{1}^{2}\exp\left(-i\Delta k\xi \right)=0. \end{array}$$ (25)

Let us raise to the second power the expressions in brackets and then neglect the terms with the second order derivative on *ξ* − coordinate:

$$\begin{array}{c} (2ik_{1}\frac{\partial }{\partial \xi }-2i\beta_{1}k_{1}\frac{\partial }{\partial \tau }-2\beta_{1}\frac{\partial^{2}}{\partial \xi \partial \tau }+\frac{\partial^{2}}{\partial x^{2}})A_{1}+\\ +(2ik_{1}\beta_{1}\frac{\partial }{\partial \tau }-k_{1}\beta_{2}\frac{\partial^{2}}{\partial \tau^{2}}-\beta_{1}^{2}\frac{\partial^{2}}{\partial \tau^{2}}-i\beta_{1}\beta_{2}\frac{\partial^{3}}{\partial \tau^{3}})A_{1}+\\ +\frac{4\pi {\left(\omega_{o}\right)}^{2}\chi^{\left(2\right)}}{c^{2}}(1+\frac{i}{\omega_{o}}\frac{\partial }{\partial \tau }{)}^{2}A_{1}^{*}A_{2}\exp\left(i\Delta k\xi \right)=0, \end{array}$$ (26)

$$\begin{array}{c} (2ik_{2}+\frac{\partial }{\partial \xi }-2i\beta_{1}k_{2}\frac{\partial }{\partial \tau }-2\beta_{1}\frac{\partial^{2}}{\partial \xi \partial \tau }+\frac{\partial^{2}}{\partial x^{2}})A_{2}+\\ +(k_{2}^{2}+2ik_{2}s_{1}\frac{\partial }{\partial \tau }-k_{2}s_{2}\frac{\partial^{2}}{\partial \tau^{2}}-s_{1}^{2}\frac{\partial^{2}}{\partial \tau^{2}}-is_{1}s_{2}\frac{\partial^{3}}{\partial \tau^{3}})A_{2}+\\ +\frac{2\pi {\left(2\omega_{o}\right)}^{2}\chi^{\left(2\right)}}{c^{2}}(1+\frac{i}{2\omega_{o}}\frac{\partial }{\partial \tau }{)}^{2}A_{1}^{2}\exp\left(-i\Delta k\xi \right)=0. \end{array}$$ (27)

By grouping the terms in (26) and (27) and dividing them by 2*ik*_*j*_, *j* = 1, 2, we obtain:

$$\begin{array}{c} (1+\frac{i\beta_{1}}{k_{1}}\frac{\partial }{\partial \tau })\frac{\partial A_{1}}{\partial \xi }+\frac{i\beta_{2}}{2}(1+\frac{i\beta_{1}}{k_{1}}\frac{\partial }{\partial \tau })\frac{\partial^{2}A_{1}}{\partial \tau^{2}}-\frac{i}{2k_{1}}\frac{\partial^{2}A_{1}}{\partial x^{2}}-\\ -i\frac{2\pi \omega_{o}\chi^{\left(2\right)}}{cn_{\omega }}(1+\frac{i}{\omega_{o}}\frac{\partial }{\partial \tau }{)}^{2}A_{1}^{*}A_{2}\exp\left(i\Delta k\xi \right)=0, \end{array}$$ (28)

$$\begin{array}{c} (1+\frac{i\beta_{1}}{k_{2}}\frac{\partial }{\partial \tau })\frac{\partial A_{2}}{\partial \xi }-\left(\beta_{1}-s_{1}\right)(1+\frac{i}{2}(\frac{\beta_{1}+s_{1}}{k_{2}})\frac{\partial }{\partial \tau })\frac{\partial A_{2}}{\partial \tau }+\\ +\frac{is_{2}}{2}(1+i\frac{s_{1}}{k_{2}}\frac{\partial }{\partial \tau })\frac{\partial^{2}A_{2}}{\partial \tau^{2}}-\frac{i}{2k_{2}}\frac{\partial^{2}A_{2}}{\partial x^{2}}-\\ -i\frac{2\pi \omega_{o}\chi^{\left(2\right)}}{cn_{2\omega }}(1+\frac{i}{2\omega_{o}}\frac{\partial }{\partial \tau }{)}^{2}A_{1}^{2}\exp\left(-i\Delta k\xi \right)=0. \end{array}$$ (29)

The set of Eqs (28) and (29) describes the SHG process for the pulse with super-short duration, which is about a few femtoseconds.

Further reduction of these equations is a consequence of the following assumptions.

1. The group velocities of pulses and their phase velocities differ insignificantly:
  $$\begin{array}{c} |\beta_{1}-\frac{k_{1}}{\omega_{o}}|\ll 1, \end{array}$$ (30)
  $$\begin{array}{c} |s_{1}-\frac{k_{2}}{2\omega_{o}}|\ll 1. \end{array}$$ (31)
2. Difference between inverse values of the phase velocities of waves is many times less than unity:
  $$\begin{array}{c} |\frac{k_{2}}{2\omega_{o}}-\frac{k_{1}}{\omega_{o}}|=|\frac{\Delta k}{2\omega_{o}}|\ll 1. \end{array}$$ (32)

As a result, (28) written with respect to the wave for basic frequency *ω*_*o*_, reduces to

$$\begin{array}{c} (1+\frac{i}{\omega_{o}}\frac{\partial }{\partial \tau })(\frac{\partial A_{1}}{\partial \xi }+\frac{i\beta_{2}}{2}\frac{\partial^{2}A_{1}}{\partial t^{2}})-\\ -\frac{i}{2k_{1}}\frac{\partial^{2}A_{1}}{\partial x^{2}}-\frac{2\pi \omega_{o}\chi^{\left(2\right)}i}{cn_{\omega }}(1+\frac{i}{\omega_{o}}\frac{\partial }{\partial \tau }{)}^{2}A_{1}^{*}A_{1}\exp\left(i\Delta k\xi \right)=0, \end{array}$$ (33)

and (29) takes the following form:

$$\begin{array}{c} (1+\frac{i}{2\omega_{o}}\frac{\partial }{\partial \tau })(\frac{\partial A_{2}}{\partial \xi }-\left(\beta_{1}-s_{1}\right)\frac{\partial A_{2}}{\partial t}+\frac{is_{2}}{2}\frac{\partial^{2}A_{2}}{\partial t^{2}})-\\ -\frac{i}{2k_{2}}\frac{\partial^{2}A_{2}}{\partial x^{2}}-\frac{2\pi \omega_{o}\chi^{\left(2\right)}i}{cn_{2\omega }}(1+\frac{i}{2\omega_{o}}\frac{\partial }{\partial \tau }{)}^{2}A_{1}^{2}\exp\left(-i\Delta k\xi \right)=0. \end{array}$$ (34)

#### Second type of coordinate system transform

In a coordinate system moving with overage velocity

$$\begin{array}{c} \xi =z,\;\tau =t-\frac{\beta_{1}+s_{1}}{2}z, \end{array}$$ (35)

the set of Eqs (21) and (22) could be re-written as follows:

$$\begin{array}{c} (1+\frac{i(\beta_{1}+s_{1})}{2k_{1}}\frac{\partial }{\partial \tau })\frac{\partial A_{1}}{\partial \xi }+\\ +\frac{\left(\beta_{1}-s_{1}\right)}{2}(1+\frac{i(3\beta_{1}+s_{1})}{4k_{1}}\frac{\partial }{\partial \tau })\frac{\partial A_{1}}{\partial \tau }+\\ +\frac{i\beta_{2}}{2}(1+\frac{i\beta_{1}}{k_{1}}\frac{\partial }{\partial \tau })\frac{\partial^{2}A_{1}}{\partial \tau^{2}}-\frac{i}{2k_{1}}\frac{\partial^{2}A_{1}}{\partial x^{2}}-\\ -\frac{2\pi \omega_{o}\chi^{\left(2\right)}i}{cn_{\omega }}(1+\frac{i}{\omega_{o}}\frac{\partial }{\partial \tau }{)}^{2}A_{1}^{*}A_{2}\exp\left(i\Delta k\xi \right)=0, \end{array}$$ (36)

$$\begin{array}{c} (1+\frac{i(\beta_{1}+s_{1})}{2k_{2}}\frac{\partial }{\partial \tau })\frac{\partial A_{2}}{\partial \xi }-\\ -\frac{\left(\beta_{1}-s_{1}\right)}{2}(1+\frac{i(3s_{1}+\beta_{1})}{4k_{2}}\frac{\partial }{\partial \tau })\frac{\partial A_{2}}{\partial \tau }+\\ +\frac{is_{2}}{2}(1+\frac{is_{1}}{k_{2}}\frac{\partial }{\partial \tau })\frac{\partial^{2}A_{2}}{\partial \tau^{2}}-\frac{i}{2k_{2}}\frac{\partial^{2}A_{2}}{\partial x^{2}}-\\ -\frac{2\pi \omega_{o}\chi^{\left(2\right)}i}{cn_{2\omega }}(1+\frac{i}{2\omega_{o}}\frac{\partial }{\partial \tau }{)}^{2}A_{1}^{2}\exp\left(-i\Delta k\xi \right)=0. \end{array}$$ (37)

If the assumptions (30)–(32) are valid, then (36), with respect to basic frequency *ω*_*o*_, reduces to

$$\begin{array}{c} (1+\frac{i}{\omega_{o}}\frac{\partial }{\partial \tau })\frac{\partial A_{1}}{\partial \xi }+\frac{\left(\beta_{1}-s_{1}\right)}{2}(1+\frac{i}{\omega_{o}}\frac{\partial }{\partial \tau })\frac{\partial A_{1}}{\partial t}+\\ +\frac{i\beta_{2}}{2}(1+\frac{i}{\omega_{o}}\frac{\partial }{\partial \tau })\frac{\partial^{2}A_{1}}{\partial t^{2}}-\frac{i}{2k_{1}}\frac{\partial^{2}A_{1}}{\partial x^{2}}-\\ -\frac{2\pi \omega_{o}\chi^{\left(2\right)}i}{cn_{\omega }}(1+\frac{i}{\omega_{o}}\frac{\partial }{\partial \tau }{)}^{2}A_{1}^{*}A_{1}\exp\left(i\Delta k\xi \right)=0, \end{array}$$ (38)

and (37), with respect to doubled frequency 2*ω*_*o*_. takes the following form:

$$\begin{array}{c} (1+\frac{i}{2\omega_{o}}\frac{\partial }{\partial \tau })\frac{\partial A_{2}}{\partial \xi }-\frac{\left(\beta_{1}-s_{1}\right)}{2}(1+\frac{i}{2\omega_{o}}\frac{\partial }{\partial \tau })\frac{\partial A_{2}}{\partial t}+\\ +\frac{is_{2}}{2}(1+\frac{i}{2\omega_{o}}\frac{\partial }{\partial \tau })\frac{\partial^{2}A_{2}}{\partial t^{2}}-\frac{i}{2k_{2}}\frac{\partial^{2}A_{2}}{\partial x^{2}}-\\ -\frac{2\pi \omega_{o}\chi^{\left(2\right)}i}{cn_{2\omega }}(1+\frac{i}{2\omega_{o}}\frac{\partial }{\partial \tau }{)}^{2}A_{1}^{2}\exp\left(-i\Delta k\xi \right)=0. \end{array}$$ (39)

Below we use the First type of Coordinate system transform.

## Dimensionless variables and problem statement

Let us introduce dimensionless variables:

$$\begin{array}{c} \xi \to \frac{\xi }{l_{dif}},\;x\to \frac{x}{a},\;t\to \frac{t}{\tau_{p}}, \end{array}$$ (40)

$$\begin{array}{c} A_{j}\to \frac{A_{j}}{\sqrt{I_{o}}},\;\Delta k\to \Delta kl_{dif},\;l_{dif}=2k_{1}a^{2}. \end{array}$$ (41)

Above the parameter *τ*_*p*_ is a duration of the incident pulse for basic frequency *ω*_*o*_, *I*_*o*_ is its maximal intensity, the parameter *a* is a beam radius.

In new variables, the GNLSE set (33) and (34) takes the following form:

$$\begin{array}{c} (1+2i\gamma \frac{\partial }{\partial t})\frac{\partial A_{1}}{\partial \xi }+iD_{21}(1+2i\gamma \frac{\partial }{\partial t})\frac{\partial^{2}A_{1}}{\partial t^{2}}+iD\frac{\partial^{2}A_{1}}{\partial x^{2}}+\\ +i\alpha (1+2i\gamma \frac{\partial }{\partial t}{)}^{2}A_{1}^{*}A_{2}\exp\left(i\Delta k\xi \right)=0,\\ (1+i\gamma \frac{\partial }{\partial t})\frac{\partial A_{2}}{\partial \xi }+\nu (1+i\gamma \frac{\partial }{\partial t})\frac{\partial A_{2}}{\partial t}+iD_{22}(1+i\gamma \frac{\partial }{\partial t})\frac{\partial^{2}A_{2}}{\partial t^{2}}+\\ +\frac{iD}{2}\frac{\partial^{2}A_{2}}{\partial x^{2}}+i\alpha (1+i\gamma \frac{\partial }{\partial t}{)}^{2}A_{1}^{2}\exp\left(-i\Delta k\xi \right), \end{array}$$ (42)

which is considered in the following domain

$$\begin{array}{c} \left(\xi ,x,t\right)\in \Omega =\Omega_{\xi }\times \Omega_{o},\;\Omega_{\xi }=\left(0,L_{\xi }\right],\\ \Omega_{o}=\Omega_{x}\times \Omega_{t}=\left(0,L_{x}\right)\times \left(0,L_{t}\right), \end{array}$$

with the boundary conditions (BCs) for the functions *A*_*j*_(*ξ*, *x*, *t*), *j* = 1, 2:

$$\begin{array}{c} A_{j}{|}_{\begin{array}{c} x=0,L_{x}\\ (\xi ,t)\in \Omega_{\xi }\times \Omega_{t} \end{array}}=A_{j}{|}_{\begin{array}{c} t=0,L_{t}\\ (\xi ,x)\in \Omega_{\xi }\times \Omega_{x} \end{array}}=\frac{\partial A_{j}}{\partial t}{|}_{\begin{array}{c} t=0\\ (\xi ,x)\in \Omega_{\xi }\times \Omega_{x} \end{array}}=0, \end{array}$$ (43)

or

$$\begin{array}{c} A_{j}{|}_{\begin{array}{c} x=0,L_{x}\\ (\xi ,t)\in \Omega_{\xi }\times \Omega_{t} \end{array}}=A_{j}{|}_{\begin{array}{c} t=0,L_{t}\\ (\xi ,x)\in \Omega_{\xi }\times \Omega_{x} \end{array}}=\frac{\partial A_{j}}{\partial t}{|}_{\begin{array}{c} t=L_{t}\\ (\xi ,x)\in \Omega_{\xi }\times \Omega_{x} \end{array}}=0, \end{array}$$ (44)

and with the initial conditions

$$\begin{array}{c} A_{j}{|}_{\begin{array}{c} \xi =0\\ (x,t)\in \Omega_{x}\times \Omega_{t} \end{array}}=A_{oj}\left(x,t\right). \end{array}$$ (45)

Above *γ* is a parameter, which is inversely proportional to the doubled frequency 2*ω*_*o*_ and the pulse duration *τ*_*p*_:

$$\begin{array}{c} \gamma =\frac{1}{2\omega_{o}\tau_{p}}. \end{array}$$ (46)

The parameter *ν* describes group-velocity mismatch (GVM) normalized by pulse duration *τ*_*p*_:

$$\begin{array}{c} \nu =\frac{s_{1}-\beta_{1}}{\tau_{p}}l_{dif}. \end{array}$$ (47)

Parameters *D*_21_, *D*_22_ are equal to a ratio between the diffraction length of the beam and a dispersion length of the pulse for each wave:

$$\begin{array}{c} D_{21}=-\frac{l_{dif}}{l_{dis_{1}}}sign(\frac{\partial^{2}k}{\partial \omega^{2}}){|}_{\omega =\omega_{o}}=-\frac{k_{1}a^{2}}{\tau_{p}^{2}|\frac{\partial^{2}k}{\partial \omega^{2}}{|}^{-1}{|}_{\omega =\omega_{o}}}sign(\frac{\partial^{2}k}{\partial \omega^{2}}){|}_{\omega =\omega_{o}}, \end{array}$$ (48)

$$\begin{array}{c} D_{22}=-\frac{l_{dif}}{l_{dis_{2}}}sign(\frac{\partial^{2}k}{\partial \omega^{2}}){|}_{\omega =2\omega_{o}}=-\frac{k_{1}a^{2}}{\tau_{p}^{2}|\frac{\partial^{2}k}{\partial \omega^{2}}{|}^{-1}{|}_{\omega =2\omega_{o}}}sign(\frac{\partial^{2}k}{\partial \omega^{2}}){|}_{\omega =2\omega_{o}}. \end{array}$$ (49)

They characterize the second order dispersion of a wave packet. Parameter *D* is equal to unity for chosen normalization of spatial coordinate along which the laser pulse propagates. Parameter *α* describes the nonlinear coupling of waves and is defined in the following form

$$\begin{array}{c} \alpha =\frac{l_{dif}}{l_{nl}}=\frac{4\pi k_{1}a^{2}\chi^{\left(2\right)}\sqrt{I_{o}}}{cn_{\omega }}, \end{array}$$ (50)

which is written using the following assumption:

$$\begin{array}{c} \frac{\chi^{\left(2\right)}\left(\omega \right)}{n_{\omega }}=\frac{\chi^{\left(2\right)}\left(2\omega \right)}{n_{2\omega }}, \end{array}$$ (51)

which does not restrict our consideration. Let us note that if the equality (51) is not valid, then one can transform the equations with respect to new variables, in which we obtain only single coefficient (it characterizes the nonlinear coupling of waves for both equations, see [21]).

Usually, at theoretical analysis of the problem (42), the exponentially decaying functions *A*_*j*_(*ξ*, *x*, *t*), *j* = 1, 2 are used as the initial laser pulse distributions:

$$\begin{array}{c} A_{j}{|}_{\begin{array}{c} x\to \pm \infty \\ (\xi ,t)\in {\tilde{\Omega }}_{\xi }\times {\tilde{\Omega }}_{t} \end{array}}=\frac{\partial^{\left(n\right)}A_{j}}{\partial x^{n}}{|}_{\begin{array}{c} x\to \pm \infty \\ (\xi ,t)\in {\tilde{\Omega }}_{\xi }\times {\tilde{\Omega }}_{t} \end{array}}\to 0,\\ A_{j}{|}_{\begin{array}{c} t\to \pm \infty \\ (\xi ,x)\in {\tilde{\Omega }}_{\xi }\times {\tilde{\Omega }}_{x} \end{array}}=\frac{\partial^{\left(n\right)}A_{j}}{\partial t^{n}}{|}_{\begin{array}{c} t\to \pm \infty \\ (\xi ,x)\in {\tilde{\Omega }}_{\xi }\times {\tilde{\Omega }}_{x} \end{array}}\to 0, \end{array}$$ (52)

and instead of the BCs (43) and (44) the set of equations (42) is solved in the following unbounded (increasing of |*x*|, |*t*| and *z*) domain

$$\begin{array}{c} \left(\xi ,x,t\right)\in \tilde{\Omega }={\tilde{\Omega }}_{\xi }\times {\tilde{\Omega }}_{x}\times {\tilde{\Omega }}_{t},\\ {\tilde{\Omega }}_{\xi }=\left(0,+\infty \right),{\tilde{\Omega }}_{x}={\tilde{\Omega }}_{t}=\left(-\infty ,+\infty \right). \end{array}$$

Below, for definiteness, we use BCs (43). In addition, for convenience, below we use the previous notation for a longitudinal coordinate: *z* instead of *ξ*.

## Equations transform

For transforming the equations set (42) to the form, which does not contain a time derivative of the nonlinear response of medium and mixed derivatives on time and spatial coordinate, we use the following equalities:

$$\begin{array}{c} (1+ij\gamma \frac{\partial }{\partial t})g=ij\gamma \exp(\frac{it}{j\gamma })\frac{\partial }{\partial t}(g\exp(-\frac{it}{j\gamma })),\;j=1,2. \end{array}$$ (53)

Also, due to a finiteness of the initial distributions of complex amplitudes and boundedness of the laser pulse propagation distance, let us state the following additional conditions:

$$\begin{array}{c} \frac{\partial^{2}A_{j}}{\partial t^{2}}{|}_{\begin{array}{c} t=0\\ (z,x)\in \Omega_{z}\times \Omega_{x} \end{array}}=0. \end{array}$$ (54)

Using the transforms (53) and condition (54), GNLSE set (42) can be written as follows (see detailed derivation in Appendix [A]):

$$\begin{array}{c} \frac{\partial A_{1}}{\partial z}+iD_{21}\frac{\partial^{2}A_{1}}{\partial t^{2}}+\frac{D}{2\gamma }\exp(\frac{it}{2\gamma })\frac{\partial^{2}}{\partial x^{2}}(\int_{0}^{t}A_{1}\left(z,x,\eta \right)\exp(-\frac{i\eta }{2\gamma })d\eta )+\\ +i\alpha (1+2i\gamma \frac{\partial }{\partial t})\left(A_{1}^{*}A_{2}\right)\exp\left(i\Delta kz\right)=0, \end{array}$$ (55)

$$\begin{array}{c} \frac{\partial A_{2}}{\partial z}+\nu \frac{\partial A_{2}}{\partial t}+iD_{22}\frac{\partial^{2}A_{2}}{\partial t^{2}}+\frac{D}{2\gamma }\exp(\frac{it}{\gamma })\frac{\partial^{2}}{\partial x^{2}}(\int_{0}^{t}A_{2}\left(z,x,\eta \right)\exp(-\frac{i\eta }{\gamma })d\eta )+\\ +i\alpha (1+i\gamma \frac{\partial }{\partial t})A_{1}^{2}\exp\left(-i\Delta kz\right)=0. \end{array}$$ (56)

It should be stressed that these equations will be also used for writing the conservation laws.

For further reduction of the equations, let us introduce the functions:

$$\begin{array}{c} P_{j}\left(z,x,t\right)=\int_{0}^{t}A_{j}\left(z,x,\eta \right)\exp(-\frac{ij\eta }{2\gamma })d\eta ,j=1,2 \end{array}$$ (57)

or

$$\begin{array}{c} \frac{\partial P_{j}}{\partial t}=A_{j}\exp(-\frac{ijt}{2\gamma }),P_{j}{|}_{\begin{array}{c} t=0\\ (z,x)\in \Omega_{z}\times \Omega_{x} \end{array}}=0. \end{array}$$ (58)

Keeping in mind the relations (53) and (58), the set of Eqs (55) and (56) can be written as follows

$$\begin{array}{c} \frac{\partial P_{1}}{\partial z}+iD_{21}\int_{0}^{t}\frac{\partial^{2}A_{1}}{\partial \eta^{2}}\exp(-\frac{i\eta }{2\gamma })d\eta +\frac{D}{2\gamma }\int_{0}^{t}\frac{\partial^{2}P_{1}\left(z,x,\eta \right)}{\partial x^{2}}d\eta -\\ -2\alpha \gamma \exp(-\frac{it}{2\gamma })A_{1}^{*}A_{2}\exp\left(i\Delta kz\right)=0, \end{array}$$ (59)

$$\begin{array}{c} \frac{\partial P_{2}}{\partial z}+\nu \int_{0}^{t}\frac{\partial A_{2}}{\partial t}\exp(-\frac{i\eta }{\gamma })d\eta +iD_{22}\int_{0}^{t}\frac{\partial^{2}A_{2}}{\partial \eta^{2}}\exp(-\frac{i\eta }{\gamma })d\eta +\\ +\frac{D}{2\gamma }\int_{0}^{t}\frac{\partial^{2}P_{2}\left(z,x,\eta \right)}{\partial x^{2}}d\eta -\alpha \gamma \exp(-\frac{it}{\gamma })A_{1}^{2}\exp\left(-i\Delta kz\right)=0. \end{array}$$ (60)

Further, let us introduce the new functions *E*_*j*_(*z*, *x*, *t*), *j* = 1, 2 in accordance with a rule:

$$\begin{array}{c} E_{j}\left(z,x,t\right)=\int_{0}^{t}A_{j}\left(z,x,\eta \right)\exp(\frac{ij(t-\eta )}{2\gamma })d\eta , \end{array}$$ (61)

and the functions ${\tilde{E}}_{j}\left(z,x,t\right)$, *j* = 1, 2 as

$$\begin{array}{c} {\tilde{E}}_{j}=\exp(\frac{ijt}{2\gamma })\int_{0}^{t}(\int_{0}^{\eta }A_{j}\left(z,x,\tau \right)\exp(-\frac{ij\tau }{2\gamma })d\tau )d\eta =\\ =\exp(\frac{ijt}{2\gamma })\int_{0}^{t}P_{j}\left(z,x,\eta \right)d\eta . \end{array}$$ (62)

Then, (59) and (60) can be written in the following manner:

$$\begin{array}{c} \frac{\partial E_{1}}{\partial z}+iD_{21}\frac{\partial^{2}E_{1}}{\partial t^{2}}+\frac{D}{2\gamma }\frac{\partial^{2}{\tilde{E}}_{1}}{\partial x^{2}}-2\alpha \gamma A_{1}^{*}A_{2}\exp\left(i\Delta kz\right)=0,\\ \frac{\partial E_{2}}{\partial z}+\nu \frac{\partial E_{2}}{\partial t}+iD_{22}\frac{\partial^{2}E_{2}}{\partial t^{2}}+\frac{D}{2\gamma }\frac{\partial^{2}{\tilde{E}}_{2}}{\partial x^{2}}-\alpha \gamma A_{1}^{2}\exp\left(-i\Delta kz\right)=0, \end{array}$$ (63)

$$\left(z,x,t\right)\in \Omega =\Omega_{z}\times \Omega_{o}.$$

These equations should be solved together with the relaxation equations with respect to the functions *E*_*j*_(*z*, *x*, *t*), ${\tilde{E}}_{j}\left(z,x,t\right)$, *A*_*j*_(*z*, *x*, *t*):

$$\begin{array}{c} (\frac{\partial }{\partial t}-i\frac{j}{2\gamma })E_{j}=A_{j}, \end{array}$$ (64)

$$\begin{array}{c} (\frac{\partial }{\partial t}-i\frac{j}{2\gamma }{)}^{2}{\tilde{E}}_{j}=(\frac{\partial }{\partial t}-i\frac{j}{2\gamma })E_{j}=A_{j},\;j=1,2. \end{array}$$ (65)

Taking into account the BCs (43) or (43′), and initial conditions (45) for the functions *A*_*j*_, *j* = 1, 2, we obtain the following BCs with respect to the functions *E*_*j*_, ${\tilde{E}}_{j}$ on time coordinate:

$$\begin{array}{c} E_{j}{|}_{\begin{array}{c} t=0\\ (z,x)\in \Omega_{z}\times \Omega_{x} \end{array}}=\frac{\partial E_{j}}{\partial t}{|}_{\begin{array}{c} t=0\\ (z,x)\in \Omega_{z}\times \Omega_{x} \end{array}}={\tilde{E}}_{j}{|}_{\begin{array}{c} t=0\\ (z,x)\in \Omega_{z}\times \Omega_{x} \end{array}}=\frac{\partial {\tilde{E}}_{j}}{\partial t}{|}_{\begin{array}{c} t=0\\ (z,x)\in \Omega_{z}\times \Omega_{x} \end{array}}=0, \end{array}$$ (66)

$$\begin{array}{c} (\frac{\partial E_{j}}{\partial t}-i\frac{j}{2\gamma }E_{j}){|}_{\begin{array}{c} t=L_{t}\\ (z,x)\in \Omega_{z}\times \Omega_{x} \end{array}}=0. \end{array}$$ (67)

As consequence of BCs (43), we obtain the following conditions:

$$\begin{array}{c} \frac{\partial^{2}E_{j}}{\partial t^{2}}{|}_{\begin{array}{c} t=0\\ (z,x)\in \Omega_{z}\times \Omega_{x} \end{array}}=0, \end{array}$$ (68)

$$\begin{array}{c} (\frac{\partial^{2}E_{j}}{\partial t^{2}}-i\frac{j}{2\gamma }\frac{\partial E_{j}}{\partial t}){|}_{\begin{array}{c} t=L_{t}\\ (z,x)\in \Omega_{z}\times \Omega_{x} \end{array}}=0, \end{array}$$ (69)

if we solve the problem with BCs (43′).

Obviously, the BCs for these functions on *x*-coordinate are

$$\begin{array}{c} E_{j}{|}_{\begin{array}{c} x=0,L_{x}\\ (z,t)\in \Omega_{z}\times \Omega_{t} \end{array}}={\tilde{E}}_{j}{|}_{\begin{array}{c} x=0,L_{x}\\ (z,t)\in \Omega_{z}\times \Omega_{t} \end{array}}=0. \end{array}$$ (70)

The initial conditions for the functions *E*_*j*_, ${\tilde{E}}_{j}$, *j* = 1, 2 are

$$\begin{array}{c} E_{j}{|}_{\begin{array}{c} z=0\\ (x,t)\in \Omega_{x}\times \Omega_{t} \end{array}}=E_{jo}\left(x,t\right)=\int_{0}^{t}A_{jo}\left(x,\eta \right)\exp(\frac{i(t-\eta )}{j\gamma })d\eta , \end{array}$$ (71)

$$\begin{array}{c} {\tilde{E}}_{j}{|}_{\begin{array}{c} z=0\\ (x,t)\in \Omega_{x}\times \Omega_{t} \end{array}}={\tilde{E}}_{jo}\left(x,t\right)=\int_{0}^{t}E_{jo}\left(x,\eta \right)\exp(\frac{i(t-\eta )}{j\gamma })d\eta . \end{array}$$ (72)

All conditions written above will be used at the construction of the conservation laws.

## Problem invariants

Using new variables, some conservation laws can be derived.

### The conservation law of energy

**Theorem 1**. *The problem* (42) *with BCs* (43) *and initial conditions* (45) *possesses the conservation law of energy*

$$\begin{array}{c} I_{E}\left(z\right)=\int_{0}^{L_{x}}\int_{0}^{L_{t}}\left(\right|A_{1}{|}^{2}+|A_{2}{|}^{2})dxdt=const. \end{array}$$ (73)

*Proof*. Let us suppose that the functions *A*_*j*_ satisfy the conditions (43) and (54). Then, we multiply (55) and (56) by $A_{j}^{*}$, and the equations, conjugated to (55) and (56),—by *A*_*j*_. Then, we sum the obtained equations and integrate the resulted equation with respect to *x*, *t* coordinates in the domain Ω_*o*_. Thus, we obtain the following equation in Appendix [B]:

$$\begin{array}{c} \frac{d}{dz}\int_{0}^{L_{x}}\int_{0}^{L_{t}}\left(\right|A_{1}{|}^{2}+|A_{2}{|}^{2})dxdt-\\ -\frac{D}{2\gamma }\int_{0}^{L_{x}}(|\frac{\partial P_{1}}{\partial x}{|}^{2}{|}_{\begin{array}{c} t=L_{t}\\ (z,x)\in \Omega_{z}\times \Omega_{x} \end{array}}+|\frac{\partial P_{2}}{\partial x}{|}^{2}{|}_{\begin{array}{c} t=L_{t}\\ (z,x)\in \Omega_{z}\times \Omega_{x} \end{array}})dx=0. \end{array}$$ (74)

For the validity of the invariant (73) one has to show that the last integrals in (74) are equal to zero:

$$\begin{array}{c} \int_{0}^{L_{x}}|\frac{\partial P_{j}}{\partial x}{|}^{2}{|}_{\begin{array}{c} t=L_{t}\\ (z,x)\in \Omega_{z}\times \Omega_{x} \end{array}}dx=0,\;j=1,2. \end{array}$$ (75)

In fact, from (55) and (56) at the time moment *t* = *L*_*t*_ and from the BCs (54), it follows that

$$\begin{array}{c} \frac{\partial^{2}}{\partial x^{2}}(\int_{0}^{L_{t}}A_{j}\left(z,x,\eta \right)\exp(-\frac{ij\eta }{2\gamma })d\eta )=\frac{\partial^{2}P_{j}\left(z,x,L_{t}\right)}{\partial x^{2}}=0,\;j=1,2. \end{array}$$ (76)

This yields to the relations

$$\begin{array}{c} P_{j}{|}_{t=L_{t}}=C_{1j}\left(z\right)+C_{2j}\left(z\right)x,\;j=1,2. \end{array}$$ (77)

It means that the Fourier harmonic amplitudes at the frequencies $\frac{1}{\gamma }$ and $\frac{1}{2\gamma }$ vary with *x*-coordinate growing. This property occurs even if we consider the laser pulse linear propagation (in (42) the parameter *α* equals zero). Obviously, the physical reason of such harmonic amplitude evolution is absent. Therefore, the functions *C*_1*j*_(*z*), *C*_2*j*_(*z*) have to be equal zero. If *C*_1*j*_(*z*) will be equal to nonzero constant then the energy of the laser beam will increase with a propagation distance. Obviously, a physical reason of this is absent for the case under consideration. Consequently, the amplitudes of Fourier harmonics at the frequencies $\frac{1}{\gamma }$ and $\frac{1}{2\gamma }$ must be equal zero:

$$\begin{array}{c} P_{j}{|}_{\begin{array}{c} t=L_{t}\\ (z,x)\in \Omega_{z}\times \Omega_{x} \end{array}}=0. \end{array}$$ (78)

Thus, the invariant (73) is valid. In a case of the laser pulse nonlinear interaction (*α* ≠ 0), the same conditions take place.

**Corollary 1**. *The Fourier harmonics at the frequencies* $\frac{1}{\gamma }$ *and* $\frac{1}{2\gamma }$ *must be absent in the incident pulses spectra the energy conservation law preservation for the set of equations* (42):

$$\begin{array}{c} \int_{0}^{L_{t}}A_{jo}\left(x,\eta \right)\exp(-\frac{ij\eta }{2\gamma })d\eta =0,j=1,2. \end{array}$$ (79)

Let us note that if the parameter *γ* tends to zero, then the set of equations (42) reduces to the set of the NLSEs and the energy invariant (73) is valid. If the spectral harmonics $\frac{1}{\gamma }$ or $\frac{1}{2\gamma }$ are absent in the incident pulse spectrum, then these spectral harmonics will be absent in the pulse spectrum computed in any section of a medium. This statement validity is stated by the spectral invariants, which are formulated below.

### Spectral invariants

Spectral invariants describe an evolution of spectral harmonic amplitudes at the frequencies $\frac{1}{\gamma }$ and $\frac{1}{2\gamma }$ along *z*-coordinate and show that the amplitudes should not increase during the laser pulses interaction. For writing of the Spectral invariants, let us introduce the following additional conditions:

$$\begin{array}{c} \frac{\partial A_{j}}{\partial x}{|}_{\begin{array}{c} x=0,L_{x}\\ (z,t)\in \Omega_{z}\times \Omega_{t} \end{array}}=0,\;j=1,2. \end{array}$$ (80)

Actually, in laser physics the propagation distance is boundedness and the domain under consideration can be chosen in such a way that the additional conditions are valid in this domain. We note that these conditions are important only for the invariant obtaining and they are not mandatory for the problem statement. The problem could be solved without additional conditions. Obviously, instead of BCs (80) one can use BCs (52). We stress that the laser sources generate the finite beam distribution in spatial coordinates. Therefore, the statements mentioned above are valid with respect to the domain choice.

**Theorem 2**. *The problem* (42) *with BCs* (43) *and additional conditions* (78) *and* (80), *and initial conditions* (45) *possesses the spectral invariants*

$$\begin{array}{c} I_{SP_{1}}\left(z\right)=\int_{0}^{L_{x}}E_{1}\left(z,x,L_{t}\right)dx=\exp(\frac{iD_{21}z}{4\gamma^{2}})\int_{0}^{L_{x}}E_{1o}\left(x,L_{t}\right)dx, \end{array}$$ (81)

$$\begin{array}{c} I_{SP_{2}}\left(z\right)=\int_{0}^{L_{x}}E_{2}\left(z,x,L_{t}\right)dx=\exp(\frac{iz}{\gamma }(\frac{D_{22}}{\gamma }-\nu ))\int_{0}^{L_{x}}E_{2o}\left(x,L_{t}\right)dx. \end{array}$$ (82)

*Proof*. Let us consider (63) at a time moment *t* = *L*_*t*_ and integrate them with respect to *x*-coordinate:

$$\begin{array}{c} \int_{0}^{L_{x}}(\frac{\partial E_{1}}{\partial z}-\frac{iD_{21}}{4\gamma^{2}}E_{1}+\frac{D}{2\gamma }\frac{\partial^{2}{\tilde{E}}_{1}}{\partial x^{2}})dx=0, \end{array}$$ (83)

$$\begin{array}{c} \int_{0}^{L_{x}}(\frac{\partial E_{2}}{\partial z}-\frac{iD_{22}}{\gamma^{2}}E_{2}+\frac{D}{2\gamma }\frac{\partial^{2}{\tilde{E}}_{2}}{\partial x^{2}})dx=0. \end{array}$$ (84)

Taking into account the relaxation Eqs (64) and (65) at time moment *t* = *L*_*t*_

$$\begin{array}{c} (\frac{\partial E_{j}}{\partial t}-i\frac{j}{2\gamma }E_{j}){|}_{\begin{array}{c} t=L_{t}\\ (z,x)\in \Omega_{z}\times \Omega_{x} \end{array}}=0,\;j=1,2, \end{array}$$ (85)

$$\begin{array}{c} (\frac{\partial {\tilde{E}}_{j}}{\partial t}-i\frac{j}{2\gamma }{\tilde{E}}_{j}){|}_{\begin{array}{c} t=L_{t}\\ (z,x)\in \Omega_{z}\times \Omega_{x} \end{array}}=0,\;j=1,2 \end{array}$$ (86)

and additional conditions (78) we obtain:

$$\begin{array}{c} (\frac{\partial^{2}E_{j}}{\partial t^{2}}){|}_{\begin{array}{c} t=L_{t}\\ (z,x)\in \Omega_{z}\times \Omega_{x} \end{array}}=-\frac{j^{2}}{4\gamma^{2}}E_{j}{|}_{\begin{array}{c} t=L_{t}\\ (z,x)\in \Omega_{z}\times \Omega_{x} \end{array}},\;j=1,2. \end{array}$$ (87)

It is easy to see that the third terms in (83) and (84) are equal to zero:

$$\begin{array}{c} \int_{0}^{L_{x}}\frac{\partial^{2}{\tilde{E}}_{1}}{\partial x^{2}}dx=\exp(\frac{iL_{t}}{2\gamma })\int_{0}^{L_{t}}\int_{0}^{L_{x}}\frac{\partial^{2}P_{1}}{\partial x^{2}}dxd\eta =\\ =\exp(\frac{iL_{t}}{2\gamma })\int_{0}^{L_{t}}\exp(-\frac{i\eta }{2\gamma })\frac{\partial P_{1}}{\partial x}{|}_{0}^{L_{x}}d\eta . \end{array}$$ (88)

The last integral (88) equals zero due to BCs (43). Thus, we obtain the following problems:

$$\begin{array}{c} \int_{0}^{L_{x}}(\frac{\partial E_{1}}{\partial z}-\frac{iD_{21}}{4\gamma^{2}}E_{1}){|}_{\begin{array}{c} t=L_{t}\\ (z,x)\in \Omega_{z}\times \Omega_{x} \end{array}}dx=0, \end{array}$$ (89)

$$\begin{array}{c} \int_{0}^{L_{x}}(\frac{\partial E_{2}}{\partial z}-(\frac{iD_{22}}{\gamma^{2}}-\frac{i\nu }{\gamma })E_{2}){|}_{\begin{array}{c} t=L_{t}\\ (z,x)\in \Omega_{z}\times \Omega_{x} \end{array}}dx=0 \end{array}$$ (90)

with the initial conditions

$$\begin{array}{c} \int_{0}^{L_{x}}E_{1}\left(0,x,L_{t}\right)dx=\int_{0}^{L_{x}}E_{1o}\left(x,L_{t}\right)dx, \end{array}$$ (91)

$$\begin{array}{c} \int_{0}^{L_{x}}E_{2}\left(0,x,L_{t}\right)dx=\int_{0}^{L_{x}}E_{2o}\left(x,L_{t}\right)dx. \end{array}$$ (92)

Therefore, the invariants (81) and (82) can be obtained as the solutions of the problems (89)–(92).

### Hamiltonian of the problem

Let us use the following substitution for the function *A*_2_(*z*, *x*, *t*):

$$\begin{array}{c} A_{2}\left(z,x,t\right)\to A_{2}\left(z,x,t\right)\exp\left(-i\Delta kz\right). \end{array}$$ (93)

Then, the functions *E*_2_(*z*, *x*, *t*), ${\tilde{E}}_{2}\left(z,x,t\right)$ can be replaced as follows:

$$\begin{array}{c} E_{2}\left(z,x,t\right)\to E_{2}\left(z,x,t\right)\exp\left(-i\Delta kz\right), \end{array}$$ (94)

$$\begin{array}{c} {\tilde{E}}_{2}\left(z,x,t\right)\to {\tilde{E}}_{2}\left(z,x,t\right)\exp\left(-i\Delta kz\right). \end{array}$$ (95)

Using the substitution (93)– (95), the set of equations (42) can be written in the following form:

$$\begin{array}{c} (1+2i\gamma \frac{\partial }{\partial t})\frac{\partial A_{1}}{\partial z}+iD_{21}(1+2i\gamma \frac{\partial }{\partial t})\frac{\partial^{2}A_{1}}{\partial t^{2}}+iD\frac{\partial^{2}A_{1}}{\partial x^{2}}+\\ +i\alpha (1+2i\gamma \frac{\partial }{\partial t}{)}^{2}A_{1}^{*}A_{2}=0,\\ (1+i\gamma \frac{\partial }{\partial t})\frac{\partial A_{2}}{\partial z}-i\Delta k(1+i\gamma \frac{\partial }{\partial t})A_{2}+\\ +\nu (1+i\gamma \frac{\partial }{\partial t})\frac{\partial A_{2}}{\partial t}+iD_{22}(1+i\gamma \frac{\partial }{\partial t})\frac{\partial^{2}A_{2}}{\partial t^{2}}+\\ +\frac{iD}{2}\frac{\partial^{2}A_{2}}{\partial x^{2}}+i\alpha (1+i\gamma \frac{\partial }{\partial t}{)}^{2}A_{1}^{2}=0, \end{array}$$ (96)

which does not contain the terms with exp(±*i*Δ*kz*). Consequently, with respect to the substitutions (94) and (95), the set of equations (96) takes the form:

$$\frac{\partial E_{1}}{\partial z}+iD_{21}\frac{\partial^{2}E_{1}}{\partial t^{2}}+\frac{D}{2\gamma }\frac{\partial^{2}{\tilde{E}}_{1}}{\partial x^{2}}-2\alpha \gamma A_{1}^{*}A_{2}=0,$$

$$\begin{array}{c} \frac{\partial E_{2}}{\partial z}+\nu \frac{\partial E_{2}}{\partial t}+iD_{22}\frac{\partial^{2}E_{2}}{\partial t^{2}}+\frac{D}{2\gamma }\frac{\partial^{2}{\tilde{E}}_{2}}{\partial x^{2}}-i\Delta kE_{2}-\alpha \gamma A_{1}^{2}=0. \end{array}$$ (97)

**Theorem 3**. *The problem* (42) *with BCs* (43) *and additional conditions* (78), *and initial conditions* (45) *possesses a Hamiltonian*

$$\begin{array}{c} I_{H}\left(z\right)=\int_{0}^{L_{x}}\int_{0}^{L_{t}}[A_{1}^{*}(iD_{21}\frac{\partial^{2}E_{1}}{\partial t^{2}}+\frac{D}{2\gamma }\frac{\partial^{2}{\tilde{E}}_{1}}{\partial x^{2}})+A_{2}^{*}(iD_{22}\frac{\partial^{2}E_{2}}{\partial t^{2}}+\frac{D}{2\gamma }\frac{\partial^{2}{\tilde{E}}_{2}}{\partial x^{2}})+\\ +\frac{\partial E_{2}^{*}}{\partial t}\left(\nu {\tilde{E}}_{2}-i\Delta kE_{2}\right)-\alpha \gamma \left(A_{2}{\left(A_{1}^{*}\right)}^{2}+A_{2}^{*}A_{1}^{2}\right)]dxdt=const. \end{array}$$ (98)

*Proof*. Let us multiply (63) by $\frac{\partial A^{*}}{\partial z}$ and the equation, conjugated to it,—by $\frac{\partial A}{\partial z}$. Then, we sum the obtained equations and integrate the resulting expression with respect to *x*, *t* coordinates in the domain Ω_*o*_ under consideration. Then, we write an equation in the form of the sum of integrals:

$$\begin{array}{c} \int_{0}^{L_{x}}\int_{0}^{L_{t}}(\frac{\partial E_{1}}{\partial z}\frac{\partial A_{1}^{*}}{\partial z}+\frac{\partial E_{1}^{*}}{\partial z}\frac{\partial A_{1}}{\partial z})dxdt+\\ +iD_{21}\int_{0}^{L_{x}}\int_{0}^{L_{t}}(\frac{\partial^{2}E_{1}}{\partial t^{2}}\frac{\partial A_{1}^{*}}{\partial z}-\frac{\partial^{2}E_{1}^{*}}{\partial t^{2}}\frac{\partial A_{1}}{\partial z})dxdt+\\ +\frac{D}{2\gamma }\int_{0}^{L_{x}}\int_{0}^{L_{t}}(\frac{\partial^{2}{\tilde{E}}_{1}}{\partial x^{2}}\frac{\partial A_{1}^{*}}{\partial z}+\frac{\partial^{2}\tilde{E_{1}^{*}}}{\partial x^{2}}\frac{\partial A_{1}}{\partial z})dxdt+\\ +\int_{0}^{L_{x}}\int_{0}^{L_{t}}(\frac{\partial E_{2}}{\partial z}\frac{\partial A_{2}^{*}}{\partial z}+\frac{\partial E_{2}^{*}}{\partial z}\frac{\partial A_{2}}{\partial z})dxdt+\\ +iD_{22}\int_{0}^{L_{x}}\int_{0}^{L_{t}}(\frac{\partial^{2}E_{2}}{\partial t^{2}}\frac{\partial A_{2}^{*}}{\partial z}-\frac{\partial^{2}E_{2}^{*}}{\partial t^{2}}\frac{\partial A_{2}}{\partial z})dxdt+\\ +\frac{D}{2\gamma }\int_{0}^{L_{x}}\int_{0}^{L_{t}}(\frac{\partial^{2}{\tilde{E}}_{2}}{\partial x^{2}}\frac{\partial A_{2}^{*}}{\partial z}+\frac{\partial^{2}\tilde{E_{2}^{*}}}{\partial x^{2}}\frac{\partial A_{2}}{\partial z})dxdt-\\ -i\Delta k\int_{0}^{L_{x}}\int_{0}^{L_{t}}(E_{2}\frac{\partial A_{2}^{*}}{\partial z}-E_{2}^{*}\frac{\partial A_{2}}{\partial z})dxdt+\\ +\nu \int_{0}^{L_{x}}\int_{0}^{L_{t}}(\frac{\partial E_{2}}{\partial t}\frac{\partial A_{2}^{*}}{\partial z}+\frac{\partial E_{2}^{*}}{\partial t}\frac{\partial A_{2}}{\partial z})dxdt-\\ -\alpha \gamma \int_{0}^{L_{x}}\int_{0}^{L_{t}}(2A_{1}^{*}A_{2}\frac{\partial A_{1}^{*}}{\partial z}+A_{1}A_{2}^{*}\frac{\partial A_{1}}{\partial z}+A_{1}^{2}\frac{\partial A_{2}}{\partial z}+{\left(A_{1}^{*}\right)}^{2}\frac{\partial A_{2}^{*}}{\partial z})dxdt=\\ =I_{11}+iD_{21}I_{21}+\frac{D}{2\gamma }I_{31}+I_{12}+iD_{22}I_{22}+\frac{D}{2\gamma }I_{32}-\\ -i\Delta kI_{4}+\nu I_{5}-\alpha \gamma I_{6}=0. \end{array}$$ (99)

Analysis of the integrals is made in Appendix [C]. On its base, one can obtain the Hamiltonian of the problem under consideration.

**Corollary 2**. *Using the inverse transforms for* (93)–(95) *the Hamiltonian takes the following form*:

$$\begin{array}{c} I_{H}\left(z\right)=\int_{0}^{L_{x}}\int_{0}^{L_{t}}[A_{1}^{*}(iD_{21}\frac{\partial^{2}E_{1}}{\partial t^{2}}+\frac{D}{2\gamma }\frac{\partial^{2}{\tilde{E}}_{1}}{\partial x^{2}})+\\ +A_{2}^{*}(iD_{22}\frac{\partial^{2}E_{2}}{\partial t^{2}}+\frac{D}{2\gamma }\frac{\partial^{2}{\tilde{E}}_{2}}{\partial x^{2}})+\frac{\partial E_{2}^{*}}{\partial t}\left(\nu {\tilde{E}}_{2}-i\Delta kE_{2}\right)-\\ -\alpha \gamma (A_{2}\exp\left(-i\Delta kz\right){\left(A_{1}^{*}\right)}^{2}+A_{2}^{*}\exp\left(i\Delta kz\right)A_{1}^{2})]dxdt=const \end{array}$$ (100)

## Discussion

A few words about applicable range of the laser pulse parameters and a medium at which it is necessary to use the GNLSEs for a description of the process under consideration. It should be noticed that the first experiment, which demonstrated self-steepening of one pulse, was made in paper [91] for the pulse with picosecond duration propagating in optical fiber about 5 km long. The pulse, propagated this distance, possessed non-symmetrical spectrum. Therefore, a key role for application of GNLSEs plays a relation between the dispersion length of the pulse and the laser pulse propagation distance. However, the pulse self-steepening appears at short length (about a few centimeters) of a medium if the pulse duration is about 10-50 fs.

Another case of using GNLSE for the laser pulse propagation description corresponds to falling of incident laser pulse with sharp intensity distribution on a medium because an influence of a time derivative of the nonlinear response of the medium enhances many times in comparison with the Gaussian pulse propagation. However, contrast temporal nonlinear response can be induced by the laser pulse if an optical bistability occurs. As is well-known, in this case the explosive changing of the characteristics of a medium occurs. As a result, the temporal structure with strong gradient appears. Therefore, a time derivative of the nonlinear response of a medium increases its influence on the phase modulation.

A detail analysis of SHG describing in the framework of the GNLSEs has to be made using computer simulation. Nevertheless, one can suppose some characteristics features of the frequency conversion in such conditions. First, the group velocity of each of wave packets will depend on complex amplitude of another wave packet. Second, obviously that the pulse spectra will distort and become non-symmetrical. Third, the nonlinear length, that defines the distance at which the energy of base wave transfers to the energy of the wave with doubled frequency, will differ for front of the pulse and its trailing part. Fourth, because of the presence of mixed derivatives, the optical beam diffraction will depend on time moment of propagating pulse.

## Conclusions

In the framework of the SEWA approximation, we derived the GNLSEs, describing the SHG in a medium with a quadratic nonlinear response for the pulses, containing a few cycles. The main feature of the equations set concludes in a presence of the second order time derivative of the nonlinear response of a medium (dispersion of a medium nonlinear response) as well as the mixed derivatives on time and spatial coordinate.

We proposed the equations transform, which reduced these equations to the other ones, containing neither mixed derivatives of complex amplitudes nor time derivatives of the nonlinear response. These equations are more convenient for the computer simulation and for theoretical analysis of the frequency doubling process of the optical pulses, containing a few cycles.

Based on this transform we derived some conservation laws (invariants) for the SHG problem. We showed an existence of two specific frequencies (singularities in the Fourier space) inherent to the problem. They may cause an appearance of non-physical absolute instability of the problem solution if the spectral invariants are not taken into account. It should be mentioned that a presence of such singularities was also discussed in [32] at analysis of the modulation instability occurring in *χ*^(2)^-medium. We showed that the energy conservation law is valid at certain conditions on the incident pulse spectra: the spectra must not contain non-zero spectral amplitudes at two specific frequencies. Their zero-value amplitudes at the pulse propagation are provided by the spectral invariants. We derived also the Hamiltonian (the third invariant) of the problem.

All invariants mentioned above should be taken into account at least for developing of the conservative finite-difference schemes at computer simulation of the problem under consideration.

## Appendix

### A Equation transform derivation

Taking into account the transforms (53), one can write

$$\begin{array}{c} 2i\gamma \exp(\frac{it}{2\gamma })\frac{\partial }{\partial z}(\frac{\partial }{\partial t}(A_{1}\exp(\frac{-it}{2\gamma })))+\\ +iD_{21}\cdot 2i\gamma \exp(\frac{it}{2\gamma })\frac{\partial }{\partial t}(\frac{\partial^{2}A_{1}}{\partial t^{2}}\exp(-\frac{it}{2\gamma }))+iD\frac{\partial^{2}A_{1}}{\partial x^{2}}+\\ +i\alpha \cdot 2i\gamma \exp(\frac{it}{2\gamma })\frac{\partial }{\partial t}[\exp(-\frac{it}{2\gamma })(1+2i\gamma \frac{\partial }{\partial t})A_{1}^{*}A_{2}\exp\left(i\Delta kz\right)]=\\ =0, \end{array}$$ (101)

– for the first equation of the set (42), and

$$\begin{array}{c} i\gamma \exp(\frac{it}{2\gamma })\frac{\partial }{\partial z}(\frac{\partial }{\partial t}(A_{2}\exp(\frac{-it}{\gamma })))+i\nu \gamma \exp(\frac{it}{\gamma })\frac{\partial }{\partial t}(\frac{\partial A_{2}}{\partial t}\exp(-\frac{it}{\gamma }))+\\ +iD_{22}\cdot i\gamma \exp(\frac{it}{\gamma })\frac{\partial }{\partial t}(\frac{\partial^{2}A_{2}}{\partial t^{2}}\exp(-\frac{it}{\gamma }))+i\frac{D}{2}\frac{\partial^{2}A_{2}}{\partial x^{2}}+\\ +i\alpha \cdot i\gamma \exp(\frac{it}{\gamma })\frac{\partial }{\partial t}[\exp(-\frac{it}{\gamma })(1+i\gamma \frac{\partial }{\partial t})A_{1}^{2}\exp\left(-i\Delta kz\right)]=0, \end{array}$$ (102)

– for the second equation of the set (42).

Then, multiplying (101) by $\exp(-\frac{it}{2\gamma }){\left(2i\gamma \right)}^{-1}$, and (102) by $\exp(-\frac{it}{\gamma }){\left(i\gamma \right)}^{-1}$, and integrating them with respect to *t*-coordinate, we obtain:

$$\begin{array}{c} \frac{\partial }{\partial z}(A_{1}\exp(-\frac{it}{2\gamma }))+iD_{21}\frac{\partial^{2}A_{1}}{\partial t^{2}}\exp(-\frac{it}{2\gamma })-iD_{21}\frac{\partial^{2}A_{1}}{\partial t^{2}}\exp(-\frac{it}{2\gamma }){|}_{\begin{array}{c} t=0\\ (z,x)\in \Omega_{z}\times \Omega_{x} \end{array}}+\\ +\frac{D}{2\gamma }\frac{\partial^{2}}{\partial x^{2}}(\int_{0}^{t}A_{1}\left(z,x,\eta \right)\exp(-\frac{i\eta }{2\gamma })d\eta )+\\ +i\alpha \exp(-\frac{it}{2\gamma })(1+2i\gamma \frac{\partial }{\partial t})\left(A_{1}^{*}A_{2}\exp\left(i\Delta kz\right)\right)=0, \end{array}$$ (103)

$$\begin{array}{c} \frac{\partial }{\partial z}(A_{2}\exp(-\frac{it}{\gamma }))+\nu \frac{\partial A_{2}}{\partial t}\exp(-\frac{it}{\gamma })+iD_{22}\frac{\partial^{2}A_{2}}{\partial t^{2}}\exp(-\frac{it}{\gamma })-\\ -iD_{22}\frac{\partial^{2}A_{2}}{\partial t^{2}}\exp(-\frac{it}{\gamma }){|}_{\begin{array}{c} t=0\\ (z,x)\in \Omega_{z}\times \Omega_{x} \end{array}}+\\ +\frac{D}{2\gamma }\frac{\partial^{2}}{\partial x^{2}}(\int_{0}^{t}A_{2}\left(z,x,\eta \right)\exp(-\frac{i\eta }{\gamma })d\eta )+\\ +i\alpha \exp(-\frac{it}{\gamma })(1+i\gamma \frac{\partial }{\partial t})\left(A_{1}^{2}\exp\left(-i\Delta kz\right)\right)=0, \end{array}$$ (104)

correspondingly.

Due to a boundedness of the initial distribution and boundedness of the laser pulse propagation distance, let us state the following additional type of the conditions:

$$\begin{array}{c} \frac{\partial^{2}A_{j}}{\partial t^{2}}{|}_{\begin{array}{c} t=0\\ (z,x)\in \Omega_{z}\times \Omega_{x} \end{array}}=0,j=1,2. \end{array}$$ (105)

Then, (103) and (104) become the following ones:

$$\begin{array}{c} \frac{\partial }{\partial z}(A_{1}\exp(-\frac{it}{2\gamma }))+iD_{21}\frac{\partial^{2}A_{1}}{\partial t^{2}}\exp(-\frac{it}{2\gamma })+\\ +\frac{D}{2\gamma }\frac{\partial^{2}}{\partial x^{2}}(\int_{0}^{t}A_{1}\left(z,x,\eta \right)\exp(-\frac{i\eta }{2\gamma })d\eta )+\\ +i\alpha \exp(-\frac{it}{2\gamma })(1+2i\gamma \frac{\partial }{\partial t})\left(A_{1}^{*}A_{2}\exp\left(i\Delta kz\right)\right)=0, \end{array}$$ (106)

$$\begin{array}{c} \frac{\partial }{\partial z}(A_{2}\exp(-\frac{it}{\gamma }))+\nu \frac{\partial A_{2}}{\partial t}\exp(-\frac{it}{\gamma })+iD_{22}\frac{\partial^{2}A_{2}}{\partial t^{2}}\exp(-\frac{it}{\gamma })+\\ +\frac{D}{2\gamma }\frac{\partial^{2}}{\partial x^{2}}(\int_{0}^{t}A_{2}\left(z,x,\eta \right)\exp(-\frac{i\eta }{\gamma })d\eta )+\\ +i\alpha \exp(-\frac{it}{\gamma })(1+i\gamma \frac{\partial }{\partial t})\left(A_{1}^{2}\exp\left(-i\Delta kz\right)\right)=0, \end{array}$$ (107)

which contain the first derivative of the quadratic nonlinearity. Multiplying (106) by $\exp(\frac{it}{2\gamma })$, and (107) by $\exp(\frac{it}{\gamma })$, we obtain the following equations:

$$\begin{array}{c} \frac{\partial A_{1}}{\partial z}+iD_{21}\frac{\partial^{2}A_{1}}{\partial t^{2}}+\frac{D}{2\gamma }\exp(\frac{it}{2\gamma })\frac{\partial^{2}}{\partial x^{2}}(\int_{0}^{t}A_{1}\left(z,x,\eta \right)\exp(-\frac{i\eta }{2\gamma })d\eta )+\\ +i\alpha (1+2i\gamma \frac{\partial }{\partial t})\left(A_{1}^{*}A_{2}\exp\left(i\Delta kz\right)\right)=0, \end{array}$$ (108)

$$\begin{array}{c} \frac{\partial A_{2}}{\partial z}+\nu \frac{\partial A_{2}}{\partial t}+iD_{22}\frac{\partial^{2}A_{2}}{\partial t^{2}}+\\ +\frac{D}{2\gamma }\exp(\frac{it}{\gamma })\frac{\partial^{2}}{\partial x^{2}}(\int_{0}^{t}A_{2}\left(z,x,\eta \right)\exp(-\frac{i\eta }{\gamma })d\eta )+\\ +i\alpha (1+i\gamma \frac{\partial }{\partial t})\left(A_{1}^{2}\exp\left(-i\Delta kz\right)\right)=0. \end{array}$$ (109)

Let us stress that these equations are used also for writing of conservation laws.

### B Energy invariant derivation

Using (55) and (56) we obtain the equations for the functions *A*_*j*_ and $A_{j}^{*}$, *j* = 1, 2, which can be re-written as a sum of integrals:

$$\begin{array}{c} \int_{0}^{L_{x}}\int_{0}^{L_{t}}(\frac{\partial A_{1}}{\partial z}A_{1}^{*}+\frac{\partial A_{1}^{*}}{\partial z}A_{1}+\frac{\partial A_{2}}{\partial z}A_{2}^{*}+\frac{\partial A_{2}^{*}}{\partial z}A_{2})dxdt+\\ +\nu \int_{0}^{L_{x}}\int_{0}^{L_{t}}(\frac{\partial A_{2}}{\partial t}A_{2}^{*}+\frac{\partial A_{2}^{*}}{\partial t}A_{2})dxdt+\\ +iD_{21}\int_{0}^{L_{x}}\int_{0}^{L_{t}}(\frac{\partial^{2}A_{1}}{\partial t^{2}}A_{1}^{*}-\frac{\partial^{2}A_{1}^{*}}{\partial t^{2}}A_{1})dxdt+\\ +iD_{22}\int_{0}^{L_{x}}\int_{0}^{L_{t}}(\frac{\partial^{2}A_{2}}{\partial t^{2}}A_{2}^{*}-\frac{\partial^{2}A_{2}^{*}}{\partial t^{2}}A_{2})dxdt+\\ +\frac{D}{2\gamma }\int_{0}^{L_{x}}\int_{0}^{L_{t}}[\frac{\partial^{2}}{\partial x^{2}}(\int_{0}^{t}A_{1}\left(z,x,\eta \right)\exp(-\frac{i\eta }{2\gamma })d\eta )\exp(\frac{it}{2\gamma })A_{1}^{*}+\\ +\frac{\partial^{2}}{\partial x^{2}}(\int_{0}^{t}A_{1}^{*}\left(z,x,\eta \right)\exp(\frac{i\eta }{2\gamma })d\eta )\exp(-\frac{it}{2\gamma })A_{1}]dxdt+\\ +\frac{D}{2\gamma }\int_{0}^{L_{x}}\int_{0}^{L_{t}}[\frac{\partial^{2}}{\partial x^{2}}(\int_{0}^{t}A_{2}\left(z,x,\eta \right)\exp(-\frac{i\eta }{\gamma })d\eta )\exp(\frac{it}{\gamma })A_{2}^{*}+\\ +\frac{\partial^{2}}{\partial x^{2}}(\int_{0}^{t}A_{2}^{*}\left(z,x,\eta \right)\exp(\frac{i\eta }{\gamma })d\eta )\exp(-\frac{it}{\gamma })A_{2}]dxdt+\\ +i\alpha \int_{0}^{L_{x}}\int_{0}^{L_{t}}[(1+2i\gamma \frac{\partial }{\partial t})\left({\left(A_{1}^{*}\right)}^{2}A_{2}\right)\exp\left(i\Delta kz\right)-\\ -(1-2i\gamma \frac{\partial }{\partial t})\left(A_{1}^{2}A_{2}^{*}\right)\exp\left(-i\Delta kz\right)+\\ +(1+i\gamma \frac{\partial }{\partial t})\left(A_{1}^{2}A_{2}^{*}\right)\exp\left(-i\Delta kz\right)-\\ -(1-i\gamma \frac{\partial }{\partial t})\left({\left(A_{1}^{*}\right)}^{2}A_{2}\right)\exp\left(i\Delta kz\right)]dxdt=\\ =I_{1}+\nu I_{2}+iD_{21}I_{31}+iD_{22}I_{32}+\frac{D}{2\gamma }I_{41}+\frac{D}{2\gamma }I_{42}+i\alpha I_{5}. \end{array}$$ (110)

Below we discuss transforms of the integrals mentioned above. The first one contains the exact differential of functions *A*_1_, *A*_2_ intensities. Therefore, it is easy to see that the integral *I*_1_ transforms to the following expression:

$$\begin{array}{c} I_{1}=\frac{d}{dz}\int_{0}^{L_{x}}\int_{0}^{L_{t}}\left(\right|A_{1}{|}^{2}+|A_{2}{|}^{2})dxdt. \end{array}$$ (111)

Also, the integral *I*_2_ contains the time derivative of function *A*_2_ intensity:

$$\begin{array}{c} I_{2}=\int_{0}^{L_{x}}\int_{0}^{L_{t}}\frac{\partial |A_{2}{|}^{2}}{\partial t}dxdt, \end{array}$$ (112)

which equals zero, due to BCs (43). As a consequence of the integration by parts, the equalities

$$\begin{array}{c} I_{3j}=0,\;j=1,2, \end{array}$$

take place under the conditions (43) validity.

Let us show, that the integral of the nonlinear terms *I*_5_ equals zero. The terms in integral *I*_5_ without the parameter *γ* cancel each other and integral *I*_5_ can be written as

$$\begin{array}{c} I_{5}=3i\gamma \int_{0}^{L_{x}}\int_{0}^{L_{t}}\frac{\partial }{\partial t}({\left(A_{1}^{*}\right)}^{2}A_{2}\exp\left(i\Delta kz\right)+A_{1}^{2}A_{2}^{*}\exp\left(-i\Delta kz\right))dxdt=0. \end{array}$$ (113)

For the integrals *I*_41_, *I*_42_, taking into account the functions *P*_*j*_, *j* = 1, 2 definitions (57) and the BC (43), we obtain:

$$\begin{array}{c} I_{4j}=\int_{0}^{L_{x}}\int_{0}^{L_{t}}(\frac{\partial^{2}P_{j}}{\partial x^{2}}\frac{\partial P_{j}^{*}}{\partial t}+\frac{\partial^{2}P_{j}^{*}}{\partial x^{2}}\frac{\partial P_{j}}{\partial t})dxdt=\\ =-\int_{0}^{L_{x}}\int_{0}^{L_{t}}[\frac{\partial }{\partial t}(\frac{\partial P_{j}^{*}}{\partial x})\frac{\partial P_{j}}{\partial x}+\frac{\partial }{\partial t}(\frac{\partial P_{j}}{\partial x})\frac{\partial P_{j}^{*}}{\partial x}]dxdt=\\ =-\int_{0}^{L_{x}}\int_{0}^{L_{t}}\frac{\partial }{\partial t}|\frac{\partial P_{j}}{\partial x}{|}^{2}dxdt=-\int_{0}^{L_{x}}|\frac{\partial P_{j}}{\partial x}{|}^{2}{|}_{\begin{array}{c} t=L_{t}\\ (z,x)\in \Omega_{z}\times \Omega_{x} \end{array}}dx. \end{array}$$ (114)

Thus, (110) transforms to the following equation:

$$\begin{array}{c} \frac{d}{dz}\int_{0}^{L_{x}}\int_{0}^{L_{t}}\left(\right|A_{1}{|}^{2}+|A_{2}{|}^{2})dxdt-\\ -\frac{D}{2\gamma }\int_{0}^{L_{x}}(|\frac{\partial P_{1}}{\partial x}{|}^{2}{|}_{\begin{array}{c} t=L_{t}\\ (z,x)\in \Omega_{z}\times \Omega_{x} \end{array}}+|\frac{\partial P_{2}}{\partial x}{|}^{2}{|}_{\begin{array}{c} t=L_{t}\\ (z,x)\in \Omega_{z}\times \Omega_{x} \end{array}})dx=0. \end{array}$$ (115)

### C Hamiltonian derivation

As mentioned in (99), we obtain a sum of the integrals. Let us transform them. First, taking into account (64) and (78) and integrating of the *I*_1*j*_ by parts we obtain:

$$\begin{array}{c} I_{1j}=\int_{0}^{L_{x}}\int_{0}^{L_{t}}(\frac{\partial E_{j}}{\partial z}(\frac{\partial^{2}E_{j}^{*}}{\partial z\partial t}+\frac{i}{\gamma }\frac{\partial E_{j}^{*}}{\partial z})+\\ +\frac{\partial E_{j}^{*}}{\partial z}(\frac{\partial^{2}E_{j}}{\partial z\partial t}-\frac{ij}{2\gamma }\frac{\partial E_{j}}{\partial z}))dxdt=\int_{0}^{L_{x}}|\frac{\partial E_{j}}{\partial z}{|}^{2}{|}_{\begin{array}{c} t=L_{t}\\ (z,x)\in \Omega_{z}\times \Omega_{x} \end{array}}dx=\\ =\int_{0}^{L_{x}}|\frac{\partial P_{j}}{\partial z}{|}^{2}{|}_{\begin{array}{c} t=L_{t}\\ (z,x)\in \Omega_{z}\times \Omega_{x} \end{array}}dx=0,\;j=1,2. \end{array}$$ (116)

The integrals *I*_2*j*_ are transformed into the following expression:

$$\begin{array}{c} I_{2j}=\int_{0}^{L_{x}}\int_{0}^{L_{t}}(\frac{\partial^{2}E_{j}}{\partial t^{2}}\frac{\partial^{2}E_{j}^{*}}{\partial z\partial t}-\frac{\partial^{2}E_{j}^{*}}{\partial t^{2}}\frac{\partial^{2}E_{j}}{\partial z\partial t}+\\ +\frac{ij}{2\gamma }(\frac{\partial^{2}E_{j}}{\partial t^{2}}\frac{\partial E_{j}^{*}}{\partial z}+\frac{\partial^{2}E_{j}^{*}}{\partial t^{2}}\frac{\partial E_{j}}{\partial z}))dxdt=J_{21j}+\frac{ij}{2\gamma }J_{22j}. \end{array}$$ (117)

It is easy to see that the integrals *J*_21*j*_ and *J*_22*j*_ are transformed into the following integrals:

$$\begin{array}{c} J_{21j}=\int_{0}^{L_{x}}\int_{0}^{L_{t}}\frac{\partial }{\partial z}(\frac{\partial^{2}E_{j}}{\partial t^{2}}\frac{\partial E_{j}^{*}}{\partial t})dxdt, \end{array}$$ (118)

$$\begin{array}{c} J_{22j}=\int_{0}^{L_{x}}\int_{0}^{L_{t}}\frac{\partial }{\partial z}(\frac{\partial^{2}E_{j}}{\partial t^{2}}E_{j}^{*})dxdt. \end{array}$$ (119)

Thus, the integrals *I*_2*j*_ take the form:

$$\begin{array}{c} I_{2j}=\int_{0}^{L_{x}}\int_{0}^{L_{t}}\frac{\partial }{\partial z}(A_{j}^{*}\frac{\partial^{2}E_{j}}{\partial t^{2}})dxdt. \end{array}$$ (120)

The integrals *I*_3*j*_ can be written in the following way:

$$\begin{array}{c} I_{3j}=\int_{0}^{L_{x}}\int_{0}^{L_{t}}(\frac{\partial^{2}\tilde{E_{j}}}{\partial x^{2}}(\frac{\partial^{2}E_{j}^{*}}{\partial z\partial t}+\frac{ij}{2\gamma }\frac{\partial E_{j}^{*}}{\partial z})+\\ +\frac{\partial^{2}{\tilde{E}}_{j}^{*}}{\partial x^{2}}(\frac{\partial^{2}E_{j}}{\partial z\partial t}-\frac{ij}{2\gamma }\frac{\partial E_{j}}{\partial z}))dxdt=\\ =\int_{0}^{L_{x}}\int_{0}^{L_{t}}(\frac{\partial^{2}{\tilde{E}}_{j}}{\partial x^{2}}\frac{\partial^{3}{\tilde{E}}_{j}^{*}}{\partial z\partial t^{2}}+\frac{\partial^{2}{\tilde{E}}_{j}^{*}}{\partial x^{2}}\frac{\partial^{3}{\tilde{E}}_{j}}{\partial z\partial t^{2}})dxdt+\\ +\frac{2ij}{2\gamma }\int_{0}^{L_{x}}\int_{0}^{L_{t}}(\frac{\partial^{2}{\tilde{E}}_{j}}{\partial x^{2}}\frac{\partial^{2}{\tilde{E}}_{j}^{*}}{\partial z\partial t}-\frac{\partial^{2}{\tilde{E}}_{j}^{*}}{\partial x^{2}}\frac{\partial^{2}{\tilde{E}}_{j}}{\partial z\partial t})dxdt-\\ -\frac{j^{2}}{4\gamma^{2}}\int_{0}^{L_{x}}\int_{0}^{L_{t}}(\frac{\partial^{2}{\tilde{E}}_{j}}{\partial x^{2}}\frac{\partial {\tilde{E^{*}}}_{j}}{\partial z}+\frac{\partial^{2}{\tilde{E^{*}}}_{j}}{\partial x^{2}}\frac{\partial {\tilde{E}}_{j}}{\partial z})dxdt=\\ =J_{31j}+\frac{2ij}{2\gamma }J_{32j}-\frac{j^{2}}{4\gamma^{2}}J_{33j}, \end{array}$$ (121)

where

$$\begin{array}{c} J_{31j}=\int_{0}^{L_{x}}\int_{0}^{L_{t}}\frac{\partial }{\partial z}(\frac{\partial^{2}{\tilde{E}}_{j}}{\partial x^{2}}\frac{\partial^{2}{\tilde{E}}_{j}^{*}}{\partial t^{2}})dxdt, \end{array}$$ (122)

and

$$\begin{array}{c} J_{32j}=\int_{0}^{L_{x}}\int_{0}^{L_{t}}\frac{\partial }{\partial z}(\frac{\partial^{2}{\tilde{E}}_{j}}{\partial x^{2}}\frac{\partial {\tilde{E}}_{j}^{*}}{\partial t})dxdt+\int_{0}^{L_{x}}\frac{\partial^{2}{\tilde{E}}_{j}^{*}}{\partial x^{2}}\frac{\partial {\tilde{E}}_{j}}{\partial z}{|}_{\begin{array}{c} t=L_{t}\\ (z,x)\in \Omega_{z}\times \Omega_{x} \end{array}}dx. \end{array}$$ (123)

Let us note that multiplying equation, conjugated to (63), by $\frac{\partial {\tilde{E}}_{j}}{\partial z}$, and taking into account the BCs (43) and additional conditions (78), we obtain, that the second term in (123) equals zero. Thus,

$$\begin{array}{c} J_{32j}=\int_{0}^{L_{x}}\int_{0}^{L_{t}}\frac{\partial }{\partial z}(\frac{\partial^{2}{\tilde{E}}_{j}}{\partial x^{2}}\frac{\partial {\tilde{E}}_{j}^{*}}{\partial t})dxdt. \end{array}$$ (124)

The integrals *J*_33*j*_ can be written as follows

$$\begin{array}{c} J_{33j}=\int_{0}^{L_{x}}\int_{0}^{L_{t}}\frac{\partial }{\partial z}(\frac{\partial^{2}{\tilde{E}}_{j}}{\partial x^{2}}{\tilde{E}}_{j}^{*})dxdt. \end{array}$$ (125)

Also, the integral *I*_4_, by using (64) and integrating by parts, reduces to the following one:

$$\begin{array}{c} I_{4}=\int_{0}^{L_{x}}\int_{0}^{L_{t}}(E_{2}\frac{\partial A_{2}^{*}}{\partial z}-E_{2}^{*}\frac{\partial A_{2}}{\partial z})dxdt=\\ =\int_{0}^{L_{x}}\int_{0}^{L_{t}}(E_{2}\frac{\partial^{2}E_{2}^{*}}{\partial z\partial t}+\frac{\partial E_{2}^{*}}{\partial t}\frac{\partial E_{2}}{\partial z})dxdt=\\ =\int_{0}^{L_{x}}\int_{0}^{L_{t}}\frac{\partial }{\partial z}(E_{2}\frac{\partial E_{2}^{*}}{\partial t})dxdt. \end{array}$$ (126)

The integrals in *I*_5_ can be written as

$$\begin{array}{c} I_{5}=\int_{0}^{L_{x}}\int_{0}^{L_{t}}(\frac{\partial E_{2}}{\partial t}\frac{\partial A_{2}^{*}}{\partial z}+\frac{\partial E_{2}^{*}}{\partial t}\frac{\partial A_{2}}{\partial z})dxdt=\\ =\int_{0}^{L_{x}}\int_{0}^{L_{t}}(\frac{\partial E_{2}}{\partial t}\frac{\partial }{\partial z}(\frac{\partial E_{2}^{*}}{\partial t}+\frac{i}{\gamma }E_{2}^{*})+\\ +\frac{\partial E_{2}^{*}}{\partial t}\frac{\partial }{\partial z}(\frac{\partial E_{2}}{\partial t}-\frac{i}{\gamma }E_{2}))dxdt=\\ =\int_{0}^{L_{x}}\int_{0}^{L_{t}}(\frac{\partial E_{2}}{\partial t}\frac{\partial^{2}E_{2}^{*}}{\partial z\partial t}+\frac{\partial E_{2}^{*}}{\partial t}\frac{\partial^{2}E_{2}}{\partial z\partial t})dxdt-\\ -\frac{i}{\gamma }\int_{0}^{L_{x}}\int_{0}^{L_{t}}(\frac{\partial^{2}E_{2}^{*}}{\partial t}\frac{\partial^{2}E_{2}}{\partial z}-\frac{\partial^{2}E_{2}}{\partial t}\frac{\partial^{2}E_{2}^{*}}{\partial z})dxdt=\\ =I_{51}-\frac{i}{\gamma }I_{52}. \end{array}$$ (127)

Using the condition (78) and integrating by parts, one can transform the integrals *I*_51_, *I*_52_ to the form

$$\begin{array}{c} I_{51}=\int_{0}^{L_{x}}\int_{0}^{L_{t}}\frac{\partial }{\partial z}|\frac{\partial E_{2}}{\partial t}{|}^{2}dxdt, \end{array}$$ (128)

and

$$\begin{array}{c} I_{52}=\int_{0}^{L_{x}}\int_{0}^{L_{t}}\frac{\partial }{\partial z}(\frac{\partial E_{2}^{*}}{\partial t}E_{2})dxdt. \end{array}$$ (129)

Obviously, the integral *I*_6_ can be written as follows:

$$\begin{array}{c} I_{6}=\int_{0}^{L_{x}}\int_{0}^{L_{t}}(2A_{1}^{*}A_{2}\frac{\partial A_{1}^{*}}{\partial z}+A_{1}A_{2}^{*}\frac{\partial A_{1}}{\partial z}+\\ +A_{1}^{2}\frac{\partial A_{2}}{\partial z}+{\left(A_{1}^{*}\right)}^{2}\frac{\partial A_{2}^{*}}{\partial z})dxdt=\\ \int_{0}^{L_{x}}\int_{0}^{L_{t}}(A_{2}\frac{\partial {\left(A_{1}^{*}\right)}^{2}}{\partial z}+{\left(A_{1}^{*}\right)}^{2}\frac{\partial A_{2}}{\partial z}+A_{2}^{*}\frac{\partial A_{1}^{2}}{\partial z}+A_{1}^{2}\frac{\partial A_{2}^{*}}{\partial z})dxdt=\\ =\int_{0}^{L_{x}}\int_{0}^{L_{t}}\frac{\partial }{\partial z}(A_{2}{\left(A_{1}^{*}\right)}^{2}+A_{2}^{*}A_{1}^{2})dxdt. \end{array}$$ (130)

Substituting the expressions (116), (120), (121), (126), (127) and (130) into (99), we obtain the invariant (98).
